# Life course adiposity and risk of incident osteoporosis: a prospective cohort study from the UK Biobank

**DOI:** 10.3389/fendo.2026.1839419

**Published:** 2026-08-28

**Authors:** Huimin Liu, Jicun Zhu, Qiang Zhang, Jiajun Chen, Ruoyao Huang

**Affiliations:** 1 Department of Neurology, The First Affiliated Hospital of Zhengzhou University, Zhengzhou, Henan, China; 2 The First Affiliated Hospital of Zhengzhou University, Zhengzhou, China; 3 School of Nursing and Health, Zhengzhou University, Zhengzhou, China; 4 Department of Medical Services, The First Affiliated Hospital of Zhengzhou University, Zhengzhou, China; 5 Office of Hospital Administration, The First Affiliated Hospital of Zhengzhou University, Zhengzhou, China; 6 National Engineering Laboratory for Internet Medical Systems and Applications, The First Affiliated Hospital of Zhengzhou University, Zhengzhou, China

**Keywords:** birth weight, body mass index, childhood body size, life course adiposity, osteoporosis

## Abstract

**Introduction:**

Adiposity across the life course may influence osteoporosis (OP) risk, yet the relative contributions of early-life and adult adiposity, their trajectories, and underlying molecular mechanisms remain unclear.

**Methods:**

We analyzed 357,903 participants from the UK Biobank with a median follow-up of 15.7 years. Life-course adiposity was assessed via birth weight, childhood body size, and adult BMI. Associations with incident OP were evaluated using Cox models, restricted cubic splines, and mediation analyses. Developmental mismatch trajectories were constructed to examine cumulative or discordant effects of adiposity. Plasma proteomics was used to identify molecular mediators of adult BMI on OP risk.

**Results:**

During follow-up, 11,905 participants (3.3%) developed OP, predominantly women (82%). Low birth weight, thinner childhood body size, and lower adult BMI were associated with increased incident OP risk, with adult BMI showing the strongest independent effect (underweight HR = 2.41; overweight HR = 0.63; obesity HR = 0.50). Mediation analyses indicated that adult BMI partially accounted for the effects of birth weight (67.1%) and childhood body size (56.1%) on OP. Developmental mismatch analyses revealed that consistently lean or downward discordant trajectories conferred higher OP risk, whereas upward discordance and consistently high adiposity were protective. Proteomic analyses identified 2,413 BMI-associated and 422 OP-associated plasma proteins, with 95 proteins mediating the BMI-OP relationship. Protein-protein interaction and pathway enrichment analyses highlighted 22 core mediators-including ADIPOQ (25.6% mediation) and SOST (16.7%)-involved in metabolic, endocrine, immune, and bone pathways.

**Conclusion:**

Life-course adiposity shapes OP risk, with adult BMI as a key determinant and mediator of early-life influences. Proteomic analyses reveal molecular pathways linking adiposity, hormonal signaling, immune regulation, and bone metabolism, offering potential therapeutic targets. These insights advance understanding of OP pathogenesis and may inform early risk stratification and targeted prevention strategies.

## Introduction

Osteoporosis (OP), characterized by reduced bone mineral density (BMD) and compromised bone microarchitecture, is a major global health concern and a leading cause of fragility fractures in aging populations (1). With increasing life expectancy, the burden of OP-affecting more than 200 million individuals worldwide-is projected to rise substantially, with fractures of the hip, spine, and wrist contributing to morbidity, reduced quality of life, and excess mortality (2, 3). Identifying early-life and modifiable risk factors is therefore essential for developing preventive strategies.

Emerging evidence suggests that skeletal health is shaped not only by adult lifestyle and hormonal factors but also by exposures across the life course (4). In particular, adiposity at different developmental stages may exert time-specific or cumulative effects on bone metabolism. Birth weight, as a proxy for intrauterine growth, has shown inconsistent associations with adult BMD, with some studies reporting positive (5–8), null, or even inverse associations (9, 10). Mendelian randomization (MR) studies further suggest that higher genetically predicted birth weight may increase fracture risk, indicating potential causal links (10, 11). Similarly, childhood adiposity-during the critical period of peak bone mass acquisition-may promote bone strength through mechanical loading (12), yet excess fat may impair bone quality through inflammatory and hormonal pathways (13). In adulthood, higher body mass index (BMI) is generally protective against OP through skeletal loading (14, 15), but its metabolic effects may complicate this relationship. Despite these findings, few studies have comprehensively assessed how adiposity across birth, childhood, and adulthood jointly influences OP risk.

The Developmental Origins of Health and Disease (DOHaD) hypothesis posits that mismatch between early-life growth and later-life environments-such as low birth weight followed by adult obesity-may predispose individuals to chronic conditions (16–18). Recent evidence suggests similar mismatched growth trajectories may also affect skeletal outcomes (19), but their relationship with OP remains unclear.

To uncover the mechanisms linking adiposity and bone health, proteomic profiling offers a powerful approach. Circulating proteins play key roles in bone remodeling, mediating mechanical, hormonal, and inflammatory influences on the skeleton (20). Unlike traditional biomarkers, proteomic analyses enable systematic identification of molecular mediators underlying adiposity-OP associations. However, few large-scale, prospective studies have examined proteomic mediators across the life course.

To address these gaps, we leveraged data from more than 350,000 UK Biobank participants. We aimed to: examine the associations of birth weight, childhood body size, and adult BMI with incident OP; evaluate the impact of adiposity mismatch patterns across developmental stages; and identify circulating proteins that mediate the relationships between adiposity and OP. This integrative life course and proteomic approach may provide novel insights into the temporal and molecular pathways linking adiposity to bone health.

## Methods

### Study population

The UK Biobank is a large population-based cohort of >500,000 participants aged 40–69 years recruited across the UK between 2006 and 2010 (21). For the present study, we included 488,667 participants with complete data on childhood body size and adult BMI, excluding those with baseline OP (n=10,338) or missing covariates (n=120,426), yielding a final sample of 357,903. Among them, 207,071 participants with available birth weight data contributed to birth weight analyses. Ethical approval was obtained from the NHS North West Research Ethics Committee, and all participants provided written informed consent (22).

**Figure 1:**
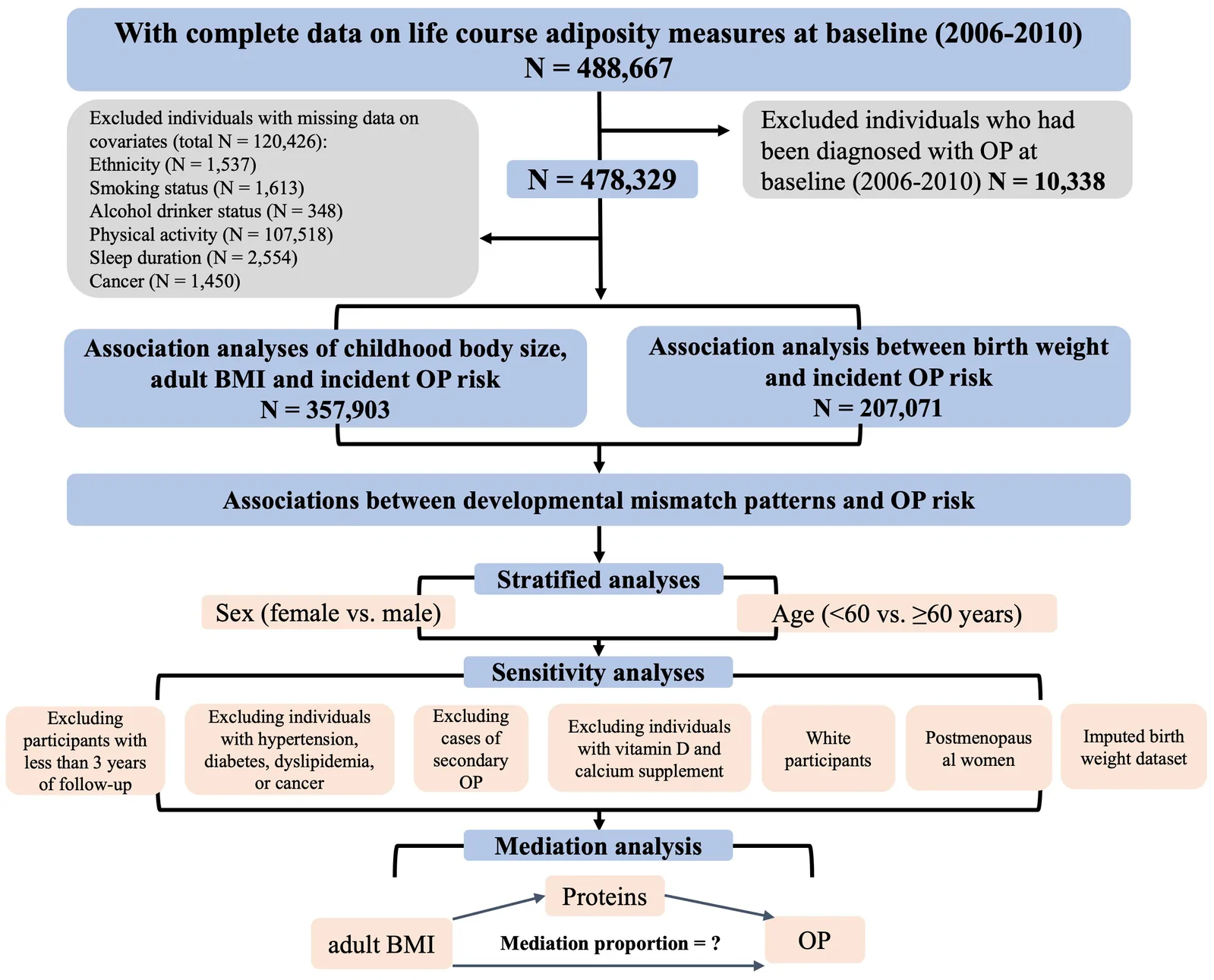
Flowchart of participant selection and overview of the analytical framework.

A flowchart illustrating participant selection and the overall analytical framework is presented in **Figure 1**.

### Incident OP

Incident OP was ascertained using UK Biobank linked health records and self-reported medical history. OP was defined according to ICD-10 codes M80, M81, and M82. The earliest recorded date across hospital inpatient records, primary care records, death registry data, and self-reported diagnoses was used as the event date. Participants with any record of OP before baseline were excluded. Follow-up extended from baseline assessment until the first OP diagnosis, death, loss to follow-up, or October 17, 2024, whichever occurred first.

### Life course adiposity and developmental mismatch patterns

Life course adiposity measures included birth weight (field ID 20022), childhood body size (field ID 1687), and adult BMI (field ID 23104). Birth weight was self-reported during verbal interviews and categorized according to WHO standards: low (<2500 g), normal (2500–3999 g), and high (≥4000 g). Birth weight information was available for 207,071 participants, while missing birth-weight data were observed in 150,832 participants. Childhood body size was assessed using a recalled measure of relative body size at approximately 10 years of age (23). Participants were asked to compare their body size during childhood with peers of the same age and sex, with responses categorized as thinner, about average, or plumper. This measure reflects perceived childhood body size rather than objectively measured childhood BMI or adiposity. Adult BMI was calculated as weight in kilograms divided by height in meters squared (kg/m^2^) and categorized as underweight (<18.5), normal weight (18.5-24.9), overweight (25.0-29.9), or obese (≥30.0).

To investigate adiposity trajectories across the life course, we created five developmental mismatch patterns based on combinations of birth weight, childhood body size, and adult BMI: Consistently lean (low birth weight, thinner childhood body size, underweight adult BMI); Consistently normal (all three within the normal range); Consistently high adiposity (high birth weight, plumper childhood body size, overweight/obese adult BMI); Upward discordance (low birth weight and thinner childhood body size, but overweight/obese adult BMI); Downward discordance (high birth weight and plumper childhood body size, but underweight adult BMI).

### Proteomic profiling

Plasma proteomic data were obtained from the UK Biobank Pharma Proteomics Project (UKB-PPP), which profiled 53,014 participants of European ancestry using the Olink Explore™ proximity extension assay platform between April 2021 and February 2022 (24). In this project, venous blood samples collected at baseline were processed into plasma and stored at −80 °C before shipment to Olink Biosciences (Sweden). Protein quantification was achieved through paired oligonucleotide-labeled antibodies followed by next-generation sequencing, and abundances were reported as normalized protein expression values after rigorous quality control and inter-plate normalization. In total, 2,923 unique proteins were measured across eight targeted panels covering cardiometabolic, inflammatory, neurological, and oncological pathways, as detailed previously (24, 25).

### Covariates

Covariates included sociodemographic factors (age, sex, ethnicity, and Index of Multiple Deprivation [IMD]), lifestyle variables (ever smoked, alcohol intake frequency, physical activity, and sleep duration), and comorbid conditions (diagnosed cancer and history of hypertension, diabetes, or dyslipidemia). Age was modeled as a continuous variable. Sex (male/female) and ethnicity (White/other) were treated as categorical variables. Socioeconomic status was assessed using the IMD, a composite area-level deprivation index incorporating income, employment, health and disability, education, barriers to housing and services, living environment, and crime domains. IMD was categorized into low, moderate, and high deprivation levels based on quartiles. Ever smoked was classified as binary variables (yes/no). Alcohol intake frequency was classified as: daily or almost daily, three or four times a week, once or twice a week, one to three times a month, special occasions only, and never. Physical activity was categorized as low, moderate, or high according to the International Physical Activity Questionnaire (IPAQ) guidelines (26). Sleep duration was dichotomized as <8 hours or ≥8 hours per night. Diagnosed cancer and histories of hypertension, diabetes, or dyslipidemia were treated as binary variables (yes/no). Data field IDs and coding schemes for covariates are provided in **Supplementary Table 1**.

### Statistical analyses

Baseline characteristics were compared between participants with and without incident OP. Continuous variables are presented as mean ± standard deviation (SD) or median (interquartile range, IQR), and categorical variables as counts (percentages).

Associations between continuous life-course adiposity measures and incident OP were examined using restricted cubic splines with knots at the 5th, 35th, 65th, and 95th percentiles (R package “rms”) to assess potential nonlinearity. Cox proportional hazards models were used to estimate hazard ratios (HRs) and 95% confidence intervals (CIs) for categorical adiposity measures and developmental mismatch patterns. Mediation analyses were conducted using the R package “mediation” to estimate the indirect associations of childhood body size and adult BMI in the relationship between birth weight and incident OP. The proportion mediated (PM) was calculated to quantify the proportion of the total association statistically explained by each mediator. E-value analyses were performed to evaluate the robustness of the observed associations to potential unmeasured confounding.

Three hierarchical Cox models were constructed: Model 1 adjusted for sociodemographic factors (age, sex, ethnicity, IMD); Model 2 additionally for lifestyle factors (smoking, alcohol intake, physical activity, sleep duration); and Model 3 further for comorbidities (hypertension, diabetes, dyslipidemia, cancer). Model 3 was considered the primary model. Stratified analyses by sex and age (<60 vs. ≥60 years) were performed using Model 3.

Sensitivity analyses (based on Model 3) included: excluding participants with <3 years follow-up; excluding those with baseline cardiometabolic diseases or cancer; excluding secondary OP; excluding vitamin D or calcium supplement users; restricting to White participants; and restricting to postmenopausal women (**Supplementary Table 1**). Missing data for birth weight were handled using complete-case analysis for the primary analyses. To evaluate the potential impact of missing birth-weight information, we performed a sensitivity analysis using multiple imputation by chained equations (MICE) with predictive mean matching. Five imputed datasets were generated, and estimates were pooled according to Rubin’s rules.

To investigate molecular mechanisms, plasma proteomic data from the UKB-PPP were analyzed. Linear regression identified BMI-associated proteins, and Cox models identified OP-associated proteins (Model 3 covariates). Overlapping proteins were considered candidate proteins in LASSO regression (“glmnet”, 10-fold cross-validation) to reduce dimensionality. Then, the significant proteins were considered candidate mediators and tested using mediation analysis with 1,000 bootstrap resamples. Only proteins with mediation effects directionally consistent with BMI were retained. Multiple testing was controlled using the Benjamini-Hochberg FDR method (FDR < 0.05).

Protein-protein interaction networks were constructed using STRING v12.0 (confidence >0.7) to identify hub proteins. KEGG pathway enrichment was performed using “clusterProfiler” (FDR < 0.05). All analyses were conducted in R version 4.5.1.

## Results

Among 357,903 participants followed for a median of 15.7 years (IQR: 15.0-16.4), 11,905 individuals (3.3%) developed incident OP. The incidence was markedly higher in women compared to men (82% vs. 18%). Participants who developed OP were generally older (median age: 62 vs. 57 years), with a higher proportion aged over 60 years. They also exhibited lower birth weight, thinner childhood body size, and lower adult BMI. Furthermore, OP cases were more likely to have a history of cancer and cardiometabolic comorbidities, including hypertension, diabetes, and dyslipidemia. Differences in ethnicity, IMD, smoking, alcohol intake frequency, physical activity, and sleep duration were also observed between OP and non-OP groups, though these were less pronounced. Collectively, these findings suggest that older, female, lower adiposity throughout life, and poorer overall health are associated with an elevated risk of developing OP. Detailed baseline characteristics by OP status are presented in **Table 1**.

**Table 1:** Baseline characteristics of participants by osteoporosis status.

Variables	N	Overall	nonOP	OP
		N = 357,903	N = 345,998	N = 11,905
Person year, median (IQR), years	357,903	15.7 (15.0, 16.4)	15.7 (15.0, 16.4)	8.7 (5.3, 11.4)
Sex, n (%)	357,903			
Female		184,878 (52)	175,115 (51)	9,763 (82)
Male		173,025 (48)	170,883 (49)	2,142 (18)
Age, median (IQR), years	357,903	57 (50, 63)	57 (49, 63)	62 (57, 66)
Age group, n (%)	357,903			
≤60		227,650 (64)	222,778 (64)	4,872 (41)
>60		130,253 (36)	123,220 (36)	7,033 (59)
Ethnicity, n (%)	357,903			
White		16,676 (5)	16,341 (5)	335 (3)
other		341,227 (95)	329,657 (95)	11,570 (97)
IMD, n (%)	357,903			
Low		126,084 (35)	122,040 (35)	4,044 (34)
Middle		121,147 (34)	117,186 (34)	3,961 (33)
High		110,672 (31)	106,772 (31)	3,900 (33)
Smoking status, n (%)	357,903			
No		142,837 (40)	138,086 (40)	4,751 (40)
Yes		215,066 (60)	207,912 (60)	7,154 (60)
Alcohol drinker status, n (%)	357,903			
Daily or almost daily		77,159 (22)	74,743 (22)	2,416 (20)
Three or four times a week		86,950 (24)	84,552 (24)	2,398 (20)
Once or twice a week		92,607 (26)	89,861 (26)	2,746 (23)
One to three times a month		39,218 (11)	37,883 (11)	1,335 (11)
Special occasions only		37,052 (10)	35,348 (10)	1,704 (14)
Never		24,917 (7)	23,611 (7)	1,306 (11)
Physical activity, n (%)	357,903			
Low		65,750 (18)	63,400 (18)	2,350 (20)
Moderate		145,598 (41)	140,623 (41)	4,975 (42)
High		146,555 (41)	141,975 (41)	4,580 (38)
Sleep duration, n (%)	357,903			
<8		226,942 (63)	219,553 (63)	7,389 (62)
>=8		130,961 (37)	126,445 (37)	4,516 (38)
Cancer, n (%)	357,903			
No		331,612 (93)	321,235 (93)	10,377 (87)
Yes		26,291 (7)	24,763 (7)	1,528 (13)
Hypertension, diabetes, or dyslipidemia, n (%)	357,903			
No		263,567 (74)	255,426 (74)	8,141 (68)
Yes		94,336 (26)	90,572 (26)	3,764 (32)
Birth weight, median (IQR), kg	207,071	3.35 (2.95, 3.66)	3.35 (2.95, 3.66)	3.23 (2.86, 3.63)
Birth weight classification, n (%)	207,071			
Low		19,656 (9)	18,754 (9)	902 (13)
Normal		158,782 (77)	153,854 (77)	4,928 (74)
High		28,633 (14)	27,761 (14)	872 (13)
Childhood body size, n (%)	357,903			
Thinner		118,924 (33)	114,870 (33)	4,054 (34)
About average		56,763 (16)	54,929 (16)	1,834 (15)
Plumper		182,216 (51)	176,199 (51)	6,017 (51)
Adult BMI continuous, median (IQR), kg/m^2^	357,903	26.6 (24.1, 29.7)	26.7 (24.2, 29.8)	25.3 (22.8, 28.5)
Adult BMI category, n (%)	357,903			
Underweight		1,664 (0)	1,457 (0)	207 (2)
Normal		118,688 (33)	113,292 (33)	5,396 (45)
Overweight		154,100 (43)	149,874 (43)	4,226 (35)
Obese		83,451 (23)	81,375 (24)	2,076 (17)

OP, osteoporosis; IQR, interquartile range; SD, standard deviation; IMD, index of multiple deprivation; BMI, body mass index; Birth weight was available for 207,071 participants and that percentages for birth-weight categories were calculated using participants with nonmissing birth-weight data as the denominator.

### Associations between life course adiposity measures and incident OP

**Figure 2:**
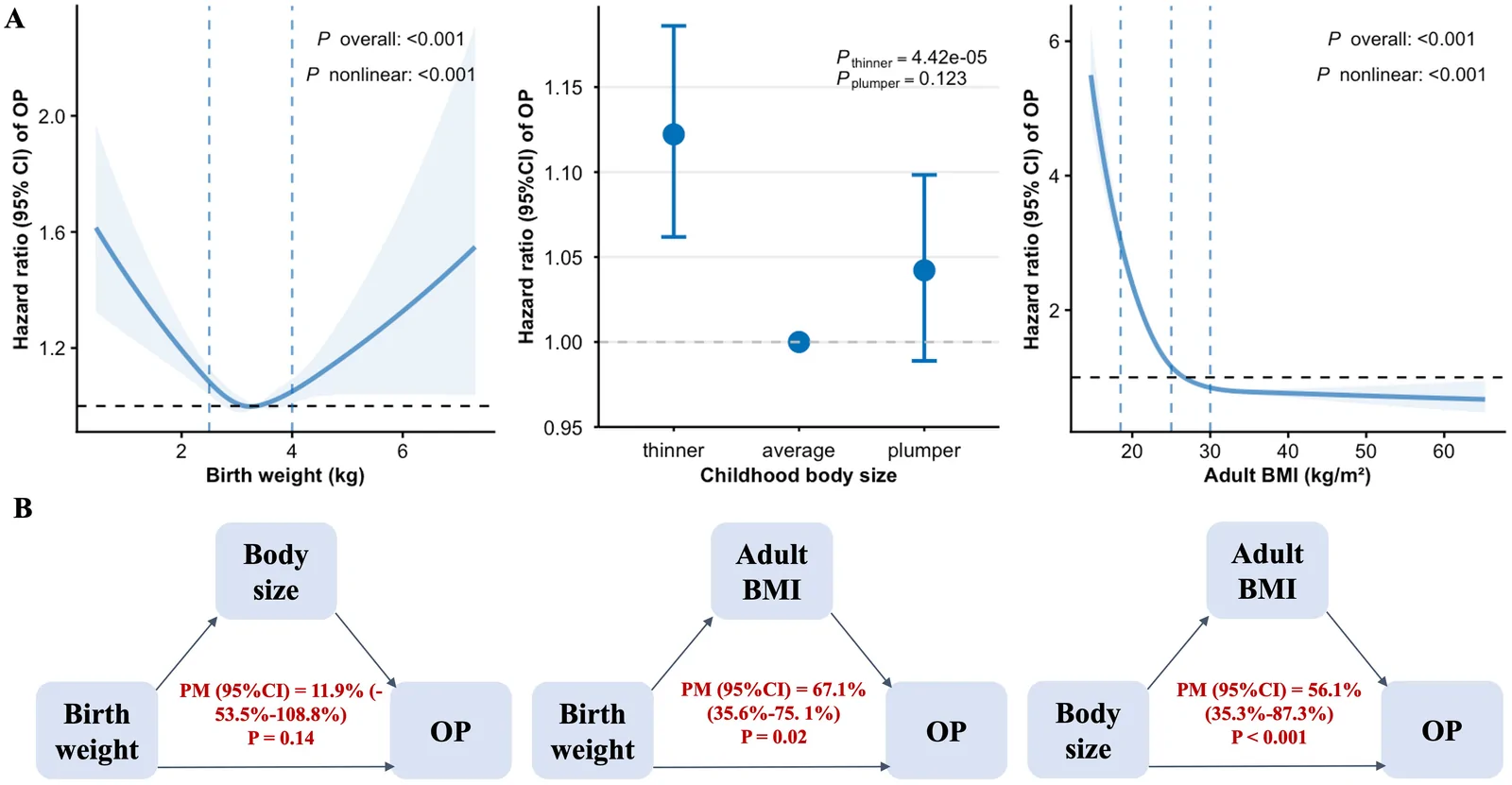
Associations between life course adiposity and incident OP. **(A)** Non-linear relationships were estimated using restricted cubic splines and linear relationships were estimated using Cox-proportional hazards regression analysis. The p values for non-linear and linear associations are listed in the figures for all outcomes. Dashed vertical lines in the left and right-hand panel represent WHO birth weight category thresholds of 2.5 kg (low to normal birth weight), 4 kg (normal to high birth weight), and BMI category thresholds of 18.5 kg/m^2^ (underweight to normal weight), 25 kg/m^2^ (normal weight to overweight), 30 kg/m^2^ (overweight to obesity). **(B)** Mediation analyses among birth weight, childhood body size, and adult BMI; PM means proportion mediated by mediator. Estimates were adjusted for age, sex, ethnicity, IMD, smoking status, alcohol consumption, physical activity, and sleep duration, hypertension, diabetes, dyslipidemia, and cancer. OP, osteoporosis; IMD, index of multiple deprivation; BMI, body mass index; PM, proportion mediated.

Dose-response relationships between continuous life course adiposity measures and OP risk were examined using restricted cubic splines. Birth weight displayed a U-shaped association, with elevated OP risk at both low and high values, while adult BMI showed an L-shaped pattern, with risk concentrated at low BMI (**Figure 2A**; **Supplementary Figure 1**).

**Figure 3:**
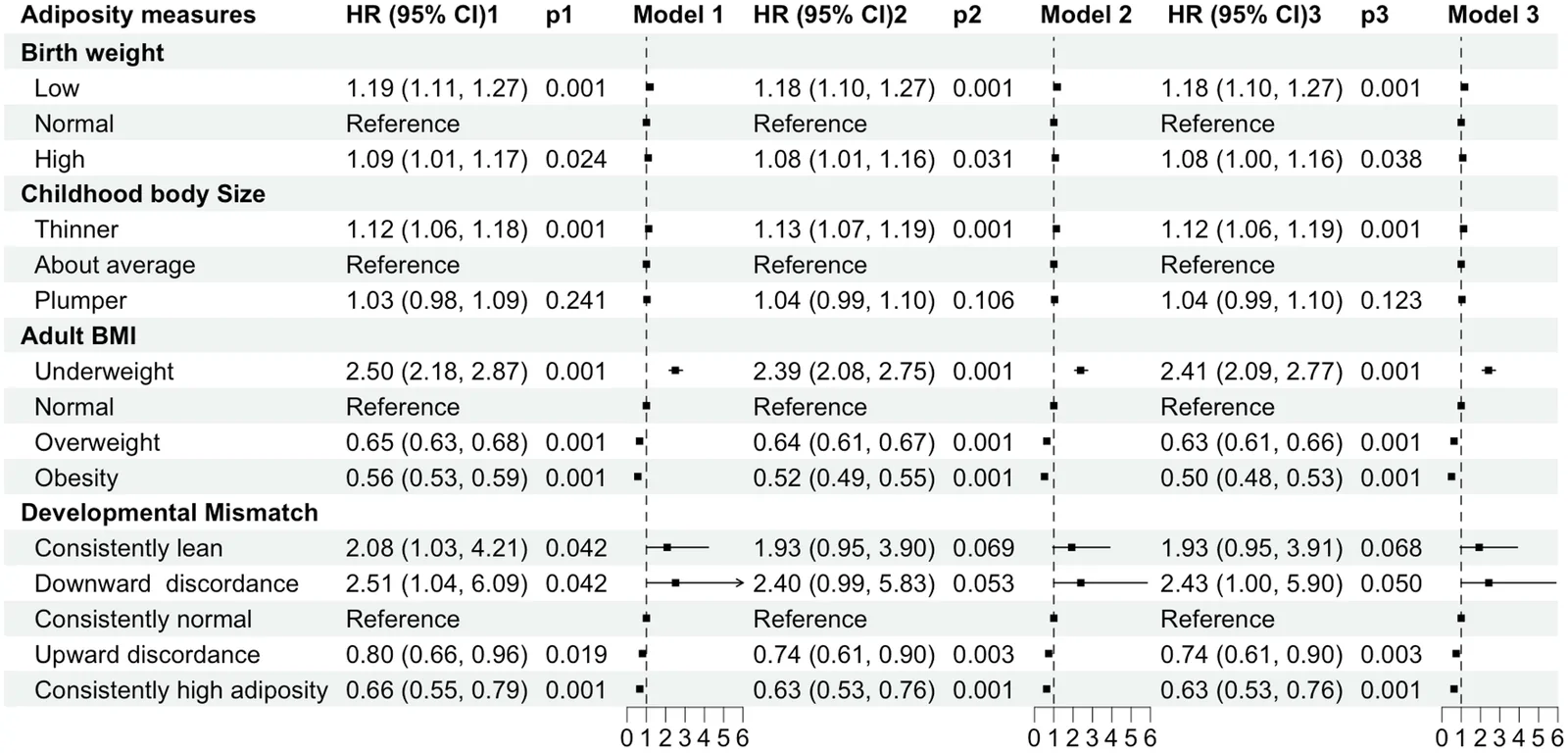
Hazard ratios for adiposity measures and incident OP. Model 1 adjusted for sociodemographic variables: age, sex, ethnicity, and IMD; Model 2 additionally adjusted for lifestyle factors: smoking status, alcohol consumption, physical activity, and sleep duration; Model 3 further adjusted for comorbid conditions: hypertension, diabetes, dyslipidemia, and cancer. Model 3 was considered the primary model for interpretation of results.

In Cox models with progressive adjustment (Models 1-3), these associations remained robust and directionally consistent (**Figure 3**). Compared with normal birth weight, both low and high birth weight were associated with increased OP risk (Model 3 HR: 1.18 [95% CI: 1.09-1.27; *P* = 0.001] for low birth weight; 1.08 [95% CI: 1.00-1.17; *P* = 0.038] for high birth weight). For childhood body size, being thinner was consistently associated with higher OP risk (HR: 1.12; 95% CI: 1.06-1.19; *P* = 0.001), whereas being plumper showed no significant association (HR: 1.04; 95% CI: 0.99-1.10; *P* = 0.123).

In contrast, adult BMI exhibited a particularly strong influence on OP risk. Underweight individuals had markedly higher risk (HR: 2.41; 95% CI: 2.09-2.77; *P* = 0.001), whereas overweight and obesity were protective (HRs: 0.63 [95% CI: 0.61-0.66] and 0.50 [95% CI: 0.48-0.53], respectively; both P = 0.001), highlighting that adult BMI not only has a substantial independent effect but also outweighs the direct effects of early-life adiposity.

Mediation analysis further reinforced this conclusion, showing adult BMI as a key mediator of early-life adiposity effects (**Figure 2B**). Adult BMI statistically explained the associations of birth weight (PM = 67.1%, *P* = 0.02) and childhood body size (PM = 56.1%, *P* < 0.001) with OP risk, whereas childhood body size did not significantly mediate the effect of birth weight (PM = 11.9%, *P* = 0.14). These findings suggest that a large proportion of the influence of early-life body size on bone health operates through adult BMI, emphasizing its central role across the life course. Sensitivity analyses using E-values indicated that moderate to strong unmeasured confounding would be required to fully explain the observed associations (**Supplementary Table 2**).

Taken together, these results indicate that while both early- and later-life adiposity contribute to OP risk, adult BMI emerges as a critical determinant, serving as both a strong independent predictor and a key mediator of earlier life influences. Given the significant influence of adiposity at multiple life stages and the mediation role of adult BMI, we further investigated whether specific life course trajectories of adiposity-captured as developmental mismatch patterns-could better explain the cumulative or discordant effects of adiposity across the lifespan on OP risk.

### Associations between developmental mismatch patterns and OP

To further examine cumulative or discordant effects of adiposity across the lifespan, participants were classified into five developmental mismatch categories based on birth weight, childhood body size, and adult BMI (**Figure 3**). Compared with individuals with consistently normal adiposity, those with consistently lean trajectories or downward discordance (higher early-life adiposity but lower adult BMI) exhibited higher OP risk (Model 3 HR: 1.93; 95% CI: 0.95-3.91; P = 0.068 for consistently lean; HR: 2.43; 95% CI: 1.00-5.90; P = 0.050 for downward discordance). In contrast, upward discordance (increase in adiposity from childhood to adulthood) and consistently high adiposity were associated with significantly reduced OP risk (Model 3 HR: 0.74 and 0.63, respectively; both P < 0.05).

These results support the developmental mismatch hypothesis, indicating that adult BMI largely determines OP risk regardless of early-life body size: higher BMI in adulthood confers protection, whereas lower BMI in adulthood increases vulnerability. Together with the mediation analysis, these findings underscore that adult BMI is the key driver of OP risk across the life course, mediating early-life influences and exerting a strong independent effect.

### Stratified analyses by sex and age

Sex- and age-stratified analyses largely corroborated the primary associations between life-course adiposity, developmental mismatch patterns, and OP risk (**Supplementary Tables 3**, **4**). Low birth weight was associated with increased OP risk mainly in females (HR = 1.21, P < 0.001) and in both younger (≤60 years, HR = 1.23) and older adults (>60 years, HR = 1.15), whereas high birth weight showed modest or non-significant effects. Thinner childhood body size predicted higher OP risk in females (HR = 1.15) and younger adults (HR = 1.20), but not in males or older individuals.

Adult BMI exhibited the strongest and most consistent associations across all subgroups. Underweight markedly elevated OP risk in both sexes (females HR = 2.27; males HR = 4.09) and across age groups (younger HR = 2.68; older HR = 2.18), while overweight and obesity were protective in all strata, with more pronounced effects in males and older adults.

Regarding developmental mismatch patterns, persistently lean trajectories and downward discordance increased OP risk in specific subgroups (particularly females and age-defined groups), whereas upward discordance and consistently high adiposity were protective across sexes and ages, especially among males and younger participants. Overall, these stratified analyses reinforce the robustness of the findings and underscore adult BMI as the dominant determinant of OP risk, while early-life adiposity and trajectory effects display subgroup-specific patterns.

### Sensitivity analysis

To assess robustness, we performed multiple sensitivity analyses, and the associations between life-course adiposity and OP risk remained materially unchanged (**Supplementary Table 5**). Low birth weight (HR 1.14-1.19) and thinner childhood body size (HR 1.11-1.15) consistently predicted higher OP risk, while adult underweight showed the strongest adverse association (HR 2.20-2.48). In contrast, overweight and obesity were stably protective across models (HR 0.46-0.65). Developmental mismatch analyses yielded concordant results: upward discordance and persistently high adiposity were associated with lower OP risk, whereas persistently lean or downward discordant patterns conferred higher risk. Overall, these findings demonstrate the stability and reliability of the primary associations.

### Proteomic pathways mediating the effect of adult BMI on OP

**Figure 4:**
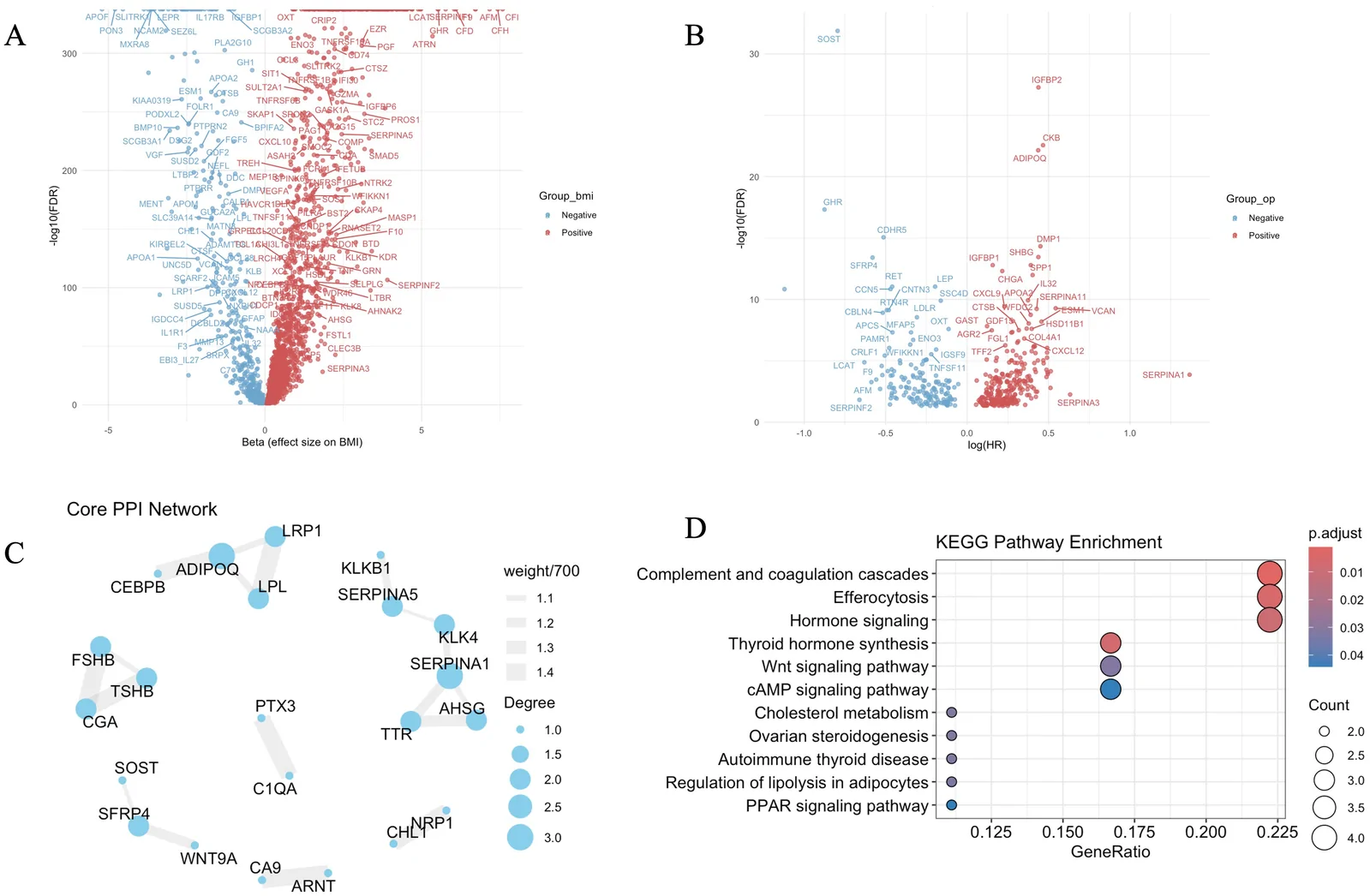
Plasma proteomic mediators linking adult BMI to osteoporosis risk. **(A)** Associations between plasma proteins and adult BMI; **(B)** Associations between plasma proteins and incident OP; **(C)** Protein-protein interaction network of core mediating proteins shared by BMI- and OP-associated proteins; **(D)** KEGG pathway enrichment of the core mediating proteins.

Linear regression identified 2,413 plasma proteins significantly associated with adult BMI (FDR < 0.05, **Figure 4A**), while Cox proportional hazards models revealed 422 proteins significantly associated with incident OP after full adjustment (FDR < 0.05, **Figure 4B**). Among these, 385 proteins were significantly associated with both BMI and OP (FDR < 0.05, **Supplementary Table 6**). Subsequent LASSO regression refined these results, yielding 172 proteins as candidate mediators (**Supplementary Table 6**). Mediation analysis demonstrated that 95 of these proteins exhibited mediation effects consistent with the BMI-OP relationship, partially accounting for the protective effect of higher BMI on OP risk, with indirect effects ranging from 0.3% to 25% of the total association (FDR < 0.05, **Supplementary Table 7**).

PPI network analysis revealed that 22 of these mediating proteins were highly interconnected, with hub nodes including SOST, ADIPOQ, KLK4, SERPINA1, and SERPINA5 (**Figure 4C**). Notably, mediation analysis demonstrated that ADIPOQ accounted for 25.6% of the adult BMI-OP association, while SOST accounted for 16.7% of this relationship. Functional enrichment analysis (KEGG) indicated that these 22 core proteins were significantly overrepresented in pathways encompassing adiposity, endocrine signaling, immune regulation, and bone metabolism (**Figure 4D**). Collectively, these findings suggest that a network of immune-inflammatory pathways, endocrine and hormonal signaling, and bone-specific metabolic processes may jointly underlie the molecular mechanism by which adult BMI influences OP risk.

## Discussion

In this large, prospective cohort study of over 350,000 participants with a median follow-up of more than 15 years, we systematically examined the associations between life-course adiposity-including birth weight, childhood body size, and adult BMI-and incident OP. Our findings provide several key insights. First, lower adiposity at any stage of life, particularly in adulthood, was consistently associated with elevated OP risk, whereas higher adult BMI exerted a robust protective effect. Second, adult BMI statistically explained the associations of both birth weight and childhood body size with OP, highlighting its central role in linking early-life adiposity to later bone health. Third, participants with persistently lean adiposity trajectories exhibited the highest OP risk, while those demonstrating upward discordance (i.e., transitioning from low to higher adiposity across the life course) had reduced risk, supporting the developmental mismatch hypothesis. Finally, mediation analyses incorporating proteomic data revealed that multiple circulating proteins-particularly SOST, ADIPOQ, KLK4, SERPINA1, and SERPINA5, accounted for the protective effect of adult BMI on OP risk through pathways integrating metabolic, hormonal, and immune regulation.

Our results align with the DOHaD hypothesis, which proposes that early-life nutritional status and body composition have long-lasting effects on adult health outcomes, including skeletal health (4, 11, 27). The observed U-shaped relationship between birth weight and OP risk suggests that both extremes may reflect adverse intrauterine environments: low birth weight may impair peak bone accrual, while high birth weight may reflect maternal hyperglycemia or excessive insulin exposure, potentially disrupting endocrine regulation of skeletal development (28). Similarly, thin body size in childhood-a marker of reduced adiposity and muscle mass-was associated with higher OP risk, consistent with evidence that inadequate early-life nutrition compromises bone mineral accumulation during critical growth periods (29, 30). By contrast, adult BMI showed the strongest association, with underweight individuals experiencing more than a twofold increase in OP risk compared with those of normal weight. Whereas overweight and obese participants had a lower risk of clinically recorded OP, consistent with previous studies showing associations between higher BMI and greater bone mineral density (31, 32). However, this finding should be interpreted cautiously. Higher BMI may increase mechanical loading and bone mineral density; meanwhile, obesity may adversely affect bone quality, promote inflammation, and increase the risk of falls and certain fracture types. Therefore, a lower incidence of clinically recorded OP does not necessarily indicate improved skeletal health or reduced fracture risk. The observed inverse association between obesity and OP may reflect a combination of biomechanical loading and endocrine factors rather than a generalized improvement in bone health.

The life-course trajectory analyses extend this framework by showing that persistently lean profiles confer the highest vulnerability to OP, likely due to cumulative deficits in nutritional, hormonal, and mechanical support for bone accrual. Conversely, individuals who transitioned from lean in early life to overweight or obese in adulthood exhibited a markedly lower OP risk, suggesting that increased energy availability and mechanical loading in adulthood may partially offset skeletal disadvantages conferred by early-life undernutrition. These findings are consistent with the developmental mismatch hypothesis, which emphasizes that incongruence between early-life exposures and adult body composition may amplify disease susceptibility (16). While prior research has largely examined mismatch in height and growth velocity (33–35), our study highlights adiposity mismatch as a critical determinant of skeletal fragility, thereby extending the developmental mismatch concept beyond stature to fat mass trajectories.

Proteomic mediation analyses provided mechanistic insight into the observed associations. Rather than representing a simple statistical correlation, the protective effect of higher BMI on OP risk appeared to be partially mediated by circulating proteins involved in metabolic, endocrine, immune, and bone pathways. Notably, ADIPOQ and SOST emerged as key mediators, accounting for 25.6% and 16.7% of the BMI-OP association, respectively. These findings are biologically plausible. ADIPOQ (adiponectin), an adipokine central to energy homeostasis, has been shown to inversely correlate with BMD, likely by promoting osteoclastogenesis and inhibiting osteoblast activity (36, 37). In contrast, SOST (sclerostin), a potent inhibitor of Wnt/β-catenin signaling, suppresses bone formation, and its regulation by adiposity and mechanical loading may represent a key biological link between fat mass and skeletal health (38, 39). KLK4 encodes kallikrein-related peptidase 4, which participates in extracellular matrix remodeling and mineralized tissue development, suggesting a possible role in bone tissue homeostasis. Beyond these single mediators, PPI network and KEGG enrichment analyses revealed that BMI-associated proteins converge on immune and inflammatory signaling, endocrine regulation, and bone remodeling pathways. This integrated view supports the notion that adiposity exerts systemic effects on bone health, mediated not only by mechanical forces but also by metabolic and inflammatory regulation.

### Strengths and limitations

This study has several notable strengths. A key strength lies in its novel integration of life-course adiposity with large-scale proteomic profiling to investigate OP risk. By adopting a life-course framework, particularly through the lens of developmental mismatch between early-life body size and adult adiposity, our study extends beyond static or cross-sectional BMI analyses. This dynamic perspective highlights how temporal changes in adiposity trajectories may shape long-term skeletal vulnerability.

Another important strength is the use of the UK Biobank and its UKB-PPP, which provides access to a large, deeply characterized cohort with extensive clinical, phenotypic, and hospital-linked health data. This rich resource enhances statistical power, minimizes selection bias, and allows longitudinal evaluation of OPincidence. Crucially, the integration of plasma proteomics adds a mechanistic layer to the epidemiological associations. By identifying proteins linked to both adult BMI and osteoporosis, we were able to highlight potential biological pathways-such as extracellular matrix remodeling, inflammatory signaling, and metabolic regulation-that may mediate the relationship between adiposity and bone health. This systems-level approach advances the understanding of biological intermediates beyond conventional biomarkers and yields novel hypotheses for translational research.

Several limitations should be acknowledged. First, birth weight and childhood body size were retrospectively reported, which may introduce recall bias and misclassification. In particular, childhood body size was based on recalled relative body size at approximately 10 years of age rather than objectively measured childhood BMI or adiposity, and the categorical nature of this measure may not fully capture the severity, duration, or timing of childhood adiposity. Second, OP was identified through routinely collected health records rather than systematic DXA screening. Therefore, undiagnosed or unrecorded cases may have been missed, and our findings should be interpreted as associations with clinically recorded OP rather than the complete burden of reduced bone mineral density. Third, birth-weight information was unavailable for a proportion of participants, which may introduce selection bias. Although multiple imputation analyses yielded results comparable to complete-case analyses, residual bias due to unmeasured determinants of birth-weight availability cannot be completely excluded. Fourth, although we adjusted for a broad range of demographic, socioeconomic, lifestyle, and clinical factors, residual confounding remains possible. In particular, information on hormone replacement therapy and glucocorticoid use was unavailable or insufficiently captured in UK Biobank. Fifth, the UK Biobank cohort is predominantly composed of individuals of White British ancestry, limiting the generalizability of our findings to other populations. Finally, although mediation analyses and E-value analyses supported the robustness of the observed associations, causal interpretation remains limited because unmeasured confounding cannot be fully excluded. Therefore, the mediation findings should be interpreted as statistical decomposition of associations rather than definitive evidence of causal pathways.

## Conclusion

These findings indicate that life-course adiposity shapes skeletal health through interconnected metabolic, hormonal, and immune mechanisms, where persistent leanness favors a catabolic bone environment and higher adult BMI promotes an anabolic state. Identification of key mediators such as ADIPOQ and SOST provides molecular insight into the adiposity-bone axis and highlights potential therapeutic targets for OP prevention.

## Data Availability

The datasets presented in this study can be found in online repositories. The names of the repository/repositories and accession number(s) can be found in the article/**Supplementary Material**.
