# Supplementary material for: Life course adiposity and risk of incident osteoporosis: a prospective cohort study from the UK Biobank
Source: Front Endocrinol (Lausanne). 2026 Aug 28;17:1839419. doi: 10.3389/fendo.2026.1839419 (PMC13561790; doi:10.3389/fendo.2026.1839419)
Supplement: Supplementary file 1 [file Supplementaryfile1.docx]

**Table S1 UK Biobank data field IDs and coding for covariates and sensitivity analysis variables**

| Variables | Field ID | Coding |
| --- | --- | --- |
| Age | 21022 | Continuous |
| Sex | 31 | 1 “Male”; 2 “female” |
| Ethnicity | 21000 | 1 “White”; 2 “Others” |
| Index of Multiple Deprivation (England) | 26410 | 1 “Lower”; 2 “Higher” |
| Index of Multiple Deprivation (Scotland) | 26427 | 1 “Lower”; 2 “Higher” |
| Index of Multiple Deprivation (Wales) | 26426 | 1 “Lower”; 2 “Higher” |
| Ever smoked | 20160 | 0 “No”; 1 “Yes” |
| Alcohol intake frequency | 1558 | 1 “Daily or almost daily”; 2 “Three or four times a week”; 3 “Once or twice a week”; 4 "One to three times a month"; 5 "Special occasions only"; 6 "Never"; |
| Physical activity | 22032 | 1 “Low”; 2 “Moderate”; 3 “High” |
| Sleep duration | 1160 | 1 “<8 hours per night”; 2 “>= 8 hours per night” |
| Cancer diagnosed by doctor | 2453 | 1 "Yes"; 0 "No" |
| History of hypertension, diabetes, or dyslipidemia | 6153 | 1 "Yes"; 0 "No" |
| Vitamin D supplements | 6155 | 1 "Yes"; 0 "No" |
| Galcium supplements | 6179 | 1 "Yes"; 0 "No" |
| Had menopause | 2724 | 1 "Yes"; 0 "No" |
| Attendance date, lost date, and death date | 53, 191, and 40000 | Month-Day-year |

**Table S2 Stratified analyses of hazard ratios for adiposity measures and incident OP by sex**

| Exposure | E-value (95% CI) |
| --- | --- |
| Birth weight |  |
| Low birth weight | 1.64 |
| High birth weight | 1.37 |
| Childhood body size |  |
| Thinner | 1.49 |
| Plumper | 1.24 |
| Adult BMI |  |
| Underweight | 4.25 |
| Overweight | 2.55 |
| Obesity | 3.41 |
| Developmental mismatch |  |
| Consistently lean | 3.27 |
| Downward discordance | 4.29 |
| Upward discordance | 2.04 |
| Consistently high adiposity | 2.55 |

E-values represent the minimum strength of association that an unmeasured confounder would need to have with both the exposure and outcome, conditional on the measured covariates, to fully explain the observed association.

**Table S3 Stratified analyses of hazard ratios for adiposity measures and incident OP by sex**

| Adiposity measures | Female |  | Male | | |  |
| --- | --- | --- | --- | --- | --- | --- |
|  | **HR (95% CI)** | **p** | | **HR (95% CI)** | ***p*** | |
| Birth weight |  |  | |  |  | |
| Low | 1.21 (1.12, 1.30) | <0.001 | | 0.93 (0.72, 1.22) | 0.614 | |
| Normal | reference |  | | reference |  | |
| High | 1.07 (0.99, 1.16) | 0.089 | | 1.11 (0.94, 1.31) | 0.235 | |
| Childhood body Size |  |  | |  |  | |
| Thinner | 1.15 (1.09, 1.23) | <0.001 | | 0.96 (0.84, 1.09) | 0.524 | |
| About average | reference |  | | reference |  | |
| Plumper | 1.07 (1.01, 1.13) | 0.028 | | 0.91 (0.80, 1.03) | 0.141 | |
| Adult BMI |  |  | |  |  | |
| Underweight | 2.27 (1.96, 2.64) | <0.001 | | 4.09 (2.76, 6.06) | <0.001 | |
| Normal | reference |  | | reference |  | |
| Overweight | 0.65 (0.63, 0.68) | <0.001 | | 0.56 (0.51, 0.62) | <0.001 | |
| Obesity | 0.48 (0.46, 0.51) | <0.001 | | 0.58 (0.52, 0.65) | <0.001 | |
| Developmental Mismatch |  |  | |  |  | |
| Consistently lean | 2.10 (1.03, 4.25) | 0.041 | | 0.69 (0.34, 1.44) | 0.325 | |
| Downward discordance | 2.55 (1.05, 6.21) | 0.039 | | 0.72 (0.39, 1.33) | 0.295 | |
| Consistently normal | reference |  | | reference |  | |
| Upward discordance | 0.81 (0.66, 0.99) | 0.042 | | 0.32 (0.16, 0.66) | 0.002 | |
| Consistently high adiposity | 0.63 (0.51, 0.77) | <0.001 | | 0.54 (0.33, 0.91) | 0.019 | |

Estimates were adjusted for age, sex, ethnicity, IMD, smoking status, alcohol consumption, physical activity, and sleep duration, hypertension, diabetes, dyslipidemia, and cancer. OP: osteoporosis;

**Table S4 Stratified analyses of hazard ratios for adiposity measures and incident OP by age**

| Adiposity measures | ≤60 |  | >60 | | |  |
| --- | --- | --- | --- | --- | --- | --- |
|  | **HR (95% CI)** | **p** | | **HR (95% CI)** | ***p*** | |
| Birth weight |  |  | |  |  | |
| Low | 1.23 (1.10, 1.37) | <0.001 | | 1.15 (1.05, 1.27) | 0.003 | |
| Normal | reference |  | | reference |  | |
| High | 1.00 (0.89, 1.12) | 0.976 | | 1.15 (1.04, 1.26) | 0.005 | |
| Childhood body Size |  |  | |  |  | |
| Thinner | 1.20 (1.10, 1.31) | <0.001 | | 1.08 (1.00, 1.16) | 0.053 | |
| About average | reference |  | | reference |  | |
| Plumper | 1.07 (0.99, 1.16) | 0.085 | | 1.02 (0.95, 1.10) | 0.54 | |
| Adult BMI |  |  | |  |  | |
| Underweight | 2.68 (2.21, 3.26) | <0.001 | | 2.18 (1.79, 2.66) | <0.001 | |
| Normal | reference |  | | reference |  | |
| Overweight | 0.58 (0.54, 0.62) | <0.001 | | 0.67 (0.63, 0.71) | <0.001 | |
| Obesity | 0.47 (0.43, 0.51) | <0.001 | | 0.53 (0.49, 0.57) | <0.001 | |
| Developmental Mismatch |  |  | |  |  | |
| Consistently lean | 2.48 (1.01, 6.07) | 0.048 | | 1.37 (0.43, 4.34) | 0.591 | |
| Downward discordance | 0.98 (0.14, 7.01) | 0.983 | | 3.97 (1.45, 10.86) | 0.007 | |
| Consistently normal | reference |  | | reference |  | |
| Upward discordance | 0.77 (0.58, 1.04) | 0.084 | | 0.73 (0.56, 0.96) | 0.025 | |
| Consistently high adiposity | 0.58 (0.44, 0.77) | <0.001 | | 0.67 (0.52, 0.87) | 0.002 | |

Estimates were adjusted for age, sex, ethnicity, IMD, smoking status, alcohol consumption, physical activity, and sleep duration, hypertension, diabetes, dyslipidemia, and cancer. OP: osteoporosis;

**Table S5 Sensitivity analyses of hazard ratios for adiposity measures and incident OP**

| Adiposity measures | Pearson year >=3 | | Without baseline hypertension, diabetes, dyslipidemia, or cancer | | Without secondary osteoporosis | | Without using vitamin D or calcium supplements | | White | | Postmenopausal women | | Imputed birth weight dataset | |
| --- | --- | --- | --- | --- | --- | --- | --- | --- | --- | --- | --- | --- | --- | --- |
|  | HR (95% CI) | *p* | HR (95% CI) | *p* | HR (95% CI) | *p* | HR (95% CI) | *p* | HR (95% CI) | *p* | HR (95% CI) | *p* | HR (95% CI) | *p* |
| Birth weight |  |  |  |  |  |  |  |  |  |  |  |  |  |  |
| Low | 1.19 (1.10, 1.28) | <0.001 | 1.14 (1.04, 1.25) | 0.004 | 1.18 (1.10, 1.27) | <0.001 | 1.18 (1.09, 1.28) | <0.001 | 1.19 (1.11, 1.28) | <0.001 | 1.17 (1.08, 1.28) | <0.001 | 1.12 (1.06, 1.19) | <0.001 |
| Normal | 1 (1, 1) |  | 1 (1, 1) |  | 1 (1, 1) |  | 1 (1, 1) |  | 1 (1, 1) |  | 1 (1, 1) |  | 1 (1, 1) |  |
| High | 1.08 (1.00, 1.17) | 0.043 | 1.04 (0.96, 1.13) | 0.356 | 1.08 (1.00, 1.16) | 0.048 | 1.06 (0.98, 1.15) | 0.122 | 1.08 (1.00, 1.16) | 0.051 | 1.09 (0.99, 1.19) | 0.071 | 1.07 (1.01, 1.13) | 0.014 |
| Childhood body Size |  |  |  |  |  |  |  |  |  |  |  |  |  |  |
| Thinner | 1.11 (1.05, 1.18) | <0.001 | 1.15 (1.08, 1.23) | <0.001 | 1.12 (1.06, 1.19) | <0.001 | 1.11 (1.04, 1.17) | 0.001 | 1.11 (1.05, 1.18) | <0.001 | 1.13 (1.05, 1.21) | 0.001 | 1.12 (1.06, 1.19) | 0.001 |
| About average | 1 (1, 1) |  | 1 (1, 1) |  | 1 (1, 1) |  | 1 (1, 1) |  | 1 (1, 1) |  | 1 (1, 1) |  | 1 (1, 1) |  |
| Plumper | 1.03 (0.97, 1.09) | 0.3 | 1.05 (0.99, 1.12) | 0.115 | 1.04 (0.99, 1.10) | 0.121 | 1.03 (0.97, 1.09) | 0.35 | 1.03 (0.98, 1.09) | 0.234 | 1.05 (0.98, 1.12) | 0.173 | 1.04 (0.99, 1.10) | 0.123 |
| Adult BMI |  |  |  |  |  |  |  |  |  |  |  |  |  |  |
| Underweight | 2.38 (2.05, 2.77) | <0.001 | 2.48 (2.14, 2.88) | <0.001 | 2.41 (2.10, 2.77) | <0.001 | 2.48 (2.14, 2.88) | <0.001 | 2.43 (2.11, 2.79) | <0.001 | 2.20 (1.85, 2.60) | <0.001 | 2.41 (2.09, 2.77) | 0.001 |
| Normal | 1 (1, 1) |  | 1 (1, 1) |  | 1 (1, 1) |  | 1 (1, 1) |  | 1 (1, 1) |  | 1 (1, 1) |  | 1 (1, 1) |  |
| Overweight | 0.64 (0.61, 0.67) | <0.001 | 0.63 (0.60, 0.66) | <0.001 | 0.63 (0.61, 0.66) | <0.001 | 0.63 (0.60, 0.66) | <0.001 | 0.63 (0.60, 0.66) | <0.001 | 0.65 (0.61, 0.68) | <0.001 | 0.63 (0.61, 0.66) | 0.001 |
| Obesity | 0.51 (0.48, 0.54) | <0.001 | 0.51 (0.47, 0.54) | <0.001 | 0.50 (0.48, 0.53) | <0.001 | 0.51 (0.47, 0.54) | <0.001 | 0.50 (0.48, 0.53) | <0.001 | 0.46 (0.43, 0.49) | <0.001 | 0.50 (0.48, 0.53) | 0.001 |
| Developmental Mismatch |  |  |  |  |  |  |  |  |  |  |  |  |  |  |
| Consistently lean | 1.96 (0.92, 4.17) | 0.081 | 2.43 (1.20, 4.95) | 0.014 | 1.94 (0.95, 3.92) | 0.067 | 2.43 (1.20, 4.95) | 0.014 | 2.03 (1.00, 4.12) | 0.049 | 1.56 (0.64, 3.81) | 0.326 | 2.01 (1.13, 3.57) | 0.017 |
| Downward discordance | 2.22 (0.83, 5.99) | 0.114 | 2.57 (1.05, 6.26) | 0.038 | 2.43 (1.00, 5.89) | 0.05 | 2.57 (1.05, 6.26) | 0.038 | 2.49 (1.03, 6.06) | 0.044 | 2.64 (1.08, 6.45) | 0.033 | 1.91 (1.02, 3.85) | 0.05 |
| Consistently normal | 1 (1, 1) |  | 1 (1, 1) |  | 1 (1, 1) |  | 1 (1, 1) |  | 1 (1, 1) |  | 1 (1, 1) |  | 1 (1, 1) |  |
| Upward discordance | 0.74 (0.60, 0.91) | 0.005 | 0.72 (0.57, 0.92) | 0.008 | 0.75 (0.61, 0.91) | 0.004 | 0.72 (0.57, 0.92) | 0.008 | 0.74 (0.61, 0.91) | 0.003 | 0.69 (0.55, 0.88) | 0.002 | 0.68 (0.58, 0.80) | <0.001 |
| Consistently high adiposity | 0.65 (0.53, 0.79) | <0.001 | 0.58 (0.47, 0.72) | <0.001 | 0.63 (0.52, 0.76) | <0.001 | 0.58 (0.47, 0.72) | <0.001 | 0.63 (0.52, 0.76) | <0.001 | 0.60 (0.48, 0.75) | <0.001 | 0.59 (0.51, 0.68) | <0.001 |

Estimates were adjusted for age, sex, ethnicity, IMD, smoking status, alcohol consumption, physical activity, and sleep duration, hypertension, diabetes, dyslipidemia, and cancer. OP: osteoporosis;

**Table S6 Mediators (proteins) regressed by exposures (adult BMI) and Cox regression by outcome (OP)**

| Mediators | Beta_exposure | P_exposure | FDR_exposure | HR_outcome | P_outcome | FDR_outcome | significant in LASSO regression |
| --- | --- | --- | --- | --- | --- | --- | --- |
| ACE2 | 1.73 | 0 | 0 | 1.16 | 0.002 | 0.015 | Yes |
| ACP5 | 0.91 | 0 | 0 | 1.33 | 0 | 0.002 | Yes |
| ADA2 | 0.74 | 0 | 0 | 1.28 | 0 | 0 | No |
| ADAM22 | 0.92 | 0 | 0 | 0.79 | 0.001 | 0.009 | No |
| ADAMTS13 | 0.22 | 0.014 | 0.017 | 0.73 | 0.002 | 0.02 | No |
| ADAMTS15 | 3.25 | 0 | 0 | 0.78 | 0 | 0 | Yes |
| ADAMTS8 | -1.1 | 0 | 0 | 0.85 | 0.003 | 0.027 | Yes |
| ADGRF5 | 1.09 | 0 | 0 | 1.32 | 0.004 | 0.031 | No |
| ADH4 | 1.49 | 0 | 0 | 0.89 | 0 | 0.002 | No |
| ADIPOQ | -1.68 | 0 | 0 | 1.55 | 0 | 0 | Yes |
| AFM | 7.17 | 0 | 0 | 0.56 | 0 | 0.001 | No |
| AGR2 | -0.18 | 0 | 0 | 1.16 | 0 | 0 | Yes |
| AGRP | 0.45 | 0 | 0 | 0.87 | 0.004 | 0.028 | Yes |
| AHNAK | 3.67 | 0 | 0 | 0.64 | 0 | 0 | No |
| AHNAK2 | 1.71 | 0 | 0 | 0.71 | 0.001 | 0.013 | Yes |
| AHSG | 1.68 | 0 | 0 | 0.67 | 0 | 0.001 | Yes |
| AMY2A | -1.79 | 0 | 0 | 1.16 | 0.004 | 0.031 | No |
| ANGPTL1 | -0.87 | 0 | 0 | 1.28 | 0.004 | 0.032 | Yes |
| ANGPTL3 | 0.65 | 0 | 0 | 1.29 | 0 | 0.003 | No |
| AOC3 | -1.21 | 0 | 0 | 1.24 | 0.007 | 0.048 | No |
| APCS | 4.44 | 0 | 0 | 0.63 | 0 | 0 | No |
| APOA1 | -2.15 | 0 | 0 | 1.39 | 0 | 0.004 | No |
| APOA2 | -1.72 | 0 | 0 | 1.48 | 0 | 0 | No |
| APOF | -5.21 | 0 | 0 | 1.53 | 0.001 | 0.009 | No |
| APOL1 | 0.67 | 0 | 0 | 0.86 | 0.004 | 0.029 | No |
| APOM | -2.19 | 0 | 0 | 1.28 | 0.007 | 0.046 | No |
| ARNT | -0.12 | 0.041 | 0.05 | 1.22 | 0.003 | 0.023 | Yes |
| ASAH2 | 1.23 | 0 | 0 | 0.84 | 0 | 0.001 | No |
| ATRN | 5.35 | 0 | 0 | 0.62 | 0.005 | 0.036 | No |
| BAG3 | 2.14 | 0 | 0 | 0.81 | 0 | 0.003 | Yes |
| BCHE | 3.54 | 0 | 0 | 0.63 | 0 | 0 | No |
| BGLAP | 0.23 | 0 | 0 | 1.12 | 0 | 0.004 | Yes |
| BMP10 | -2.79 | 0 | 0 | 1.63 | 0 | 0 | No |
| BPIFA2 | -0.76 | 0 | 0 | 1.08 | 0.003 | 0.023 | Yes |
| BST2 | 1.05 | 0 | 0 | 1.2 | 0 | 0 | No |
| BTD | 2.9 | 0 | 0 | 0.65 | 0.001 | 0.012 | No |
| BTN3A2 | 1.13 | 0 | 0 | 1.21 | 0.003 | 0.022 | Yes |
| C1QA | -0.32 | 0.001 | 0.001 | 1.67 | 0 | 0 | Yes |
| C3 | 2.46 | 0 | 0 | 0.8 | 0.002 | 0.015 | No |
| C7 | -0.99 | 0 | 0 | 1.63 | 0 | 0 | No |
| CA9 | -1.53 | 0 | 0 | 1.2 | 0 | 0.003 | Yes |
| CALB1 | -1.34 | 0 | 0 | 1.3 | 0 | 0 | Yes |
| CALB2 | 3.96 | 0 | 0 | 0.74 | 0 | 0.003 | No |
| CBLN4 | 0.86 | 0 | 0 | 0.6 | 0 | 0 | Yes |
| CCL11 | -0.95 | 0 | 0 | 1.27 | 0 | 0.003 | No |
| CCL17 | 0.22 | 0 | 0 | 1.08 | 0.002 | 0.02 | Yes |
| CCL20 | 0.61 | 0 | 0 | 1.09 | 0.001 | 0.013 | No |
| CCL28 | -0.81 | 0 | 0 | 1.19 | 0 | 0 | No |
| CCL3 | 1.25 | 0 | 0 | 1.12 | 0.001 | 0.013 | Yes |
| CCL7 | 1.39 | 0 | 0 | 1.13 | 0.001 | 0.011 | Yes |
| CCN5 | 2.56 | 0 | 0 | 0.62 | 0 | 0 | Yes |
| CD14 | 0.17 | 0.001 | 0.002 | 1.23 | 0 | 0.005 | No |
| CD22 | 2.15 | 0 | 0 | 0.82 | 0.001 | 0.008 | No |
| CD27 | 0.95 | 0 | 0 | 1.28 | 0 | 0.001 | No |
| CD274 | 1.01 | 0 | 0 | 1.33 | 0 | 0 | No |
| CD300LG | 0.61 | 0 | 0 | 1.3 | 0 | 0.001 | No |
| CD6 | 0.99 | 0 | 0 | 0.88 | 0.005 | 0.038 | No |
| CD74 | 2.22 | 0 | 0 | 1.29 | 0 | 0.002 | No |
| CDA | 1.56 | 0 | 0 | 0.77 | 0 | 0 | Yes |
| CDCP1 | 0.8 | 0 | 0 | 1.16 | 0.002 | 0.015 | No |
| CDHR2 | 2.09 | 0 | 0 | 0.9 | 0.001 | 0.008 | No |
| CDHR5 | 2.49 | 0 | 0 | 0.6 | 0 | 0 | Yes |
| CDON | 1.95 | 0 | 0 | 0.74 | 0.001 | 0.01 | No |
| CEACAM1 | 0.26 | 0.003 | 0.005 | 1.53 | 0 | 0.003 | No |
| CEACAM16 | 0.33 | 0 | 0 | 0.88 | 0 | 0.005 | No |
| CEACAM5 | -0.12 | 0 | 0 | 1.16 | 0 | 0.001 | No |
| CEACAM6 | -0.45 | 0 | 0 | 1.27 | 0 | 0.003 | No |
| CEBPB | 1.18 | 0 | 0 | 0.78 | 0 | 0.005 | Yes |
| CEP170 | 0.16 | 0 | 0 | 0.89 | 0.002 | 0.02 | Yes |
| CES1 | 1.59 | 0 | 0 | 0.91 | 0.002 | 0.015 | No |
| CFB | 4.19 | 0 | 0 | 0.78 | 0.003 | 0.023 | No |
| CFD | 6.1 | 0 | 0 | 0.63 | 0.001 | 0.011 | Yes |
| CFH | 7.43 | 0 | 0 | 0.63 | 0 | 0.002 | Yes |
| CFI | 7.59 | 0 | 0 | 0.64 | 0.001 | 0.01 | No |
| CFP | 4.46 | 0 | 0 | 0.63 | 0.001 | 0.006 | No |
| CGA | -0.2 | 0 | 0 | 1.19 | 0 | 0 | Yes |
| CHGA | -0.32 | 0 | 0 | 1.24 | 0 | 0 | No |
| CHGB | -2.33 | 0 | 0 | 1.31 | 0 | 0 | No |
| CHI3L1 | 0.69 | 0 | 0 | 1.15 | 0 | 0 | No |
| CHL1 | -1.71 | 0 | 0 | 1.36 | 0 | 0.001 | Yes |
| CHRDL2 | 0.61 | 0 | 0 | 0.78 | 0 | 0 | Yes |
| CKAP4 | 2.03 | 0 | 0 | 1.38 | 0 | 0.002 | No |
| CKB | -2.87 | 0 | 0 | 1.59 | 0 | 0 | No |
| CKMT1A_CKMT1B | -0.22 | 0 | 0 | 1.12 | 0.004 | 0.029 | Yes |
| CLEC11A | 0.52 | 0 | 0 | 1.16 | 0.003 | 0.026 | Yes |
| CLEC3B | 1.79 | 0 | 0 | 0.59 | 0 | 0.002 | No |
| CLEC5A | -0.81 | 0 | 0 | 1.51 | 0 | 0 | Yes |
| CLEC6A | 0.83 | 0 | 0 | 1.18 | 0.002 | 0.019 | No |
| CLSTN2 | -0.17 | 0.001 | 0.001 | 1.19 | 0.004 | 0.031 | Yes |
| CNDP1 | 1.18 | 0 | 0 | 0.77 | 0 | 0 | Yes |
| CNTN3 | 3.46 | 0 | 0 | 0.62 | 0 | 0 | No |
| COL15A1 | 4.7 | 0 | 0 | 0.63 | 0 | 0 | Yes |
| COL1A1 | -0.8 | 0 | 0 | 1.35 | 0.002 | 0.019 | Yes |
| COL4A1 | -2.49 | 0 | 0 | 1.44 | 0 | 0 | No |
| COL5A1 | 0.27 | 0 | 0 | 1.4 | 0 | 0 | No |
| COL9A1 | 0.27 | 0 | 0 | 1.22 | 0 | 0.001 | Yes |
| COMP | 1.99 | 0 | 0 | 0.72 | 0 | 0 | No |
| COPB2 | 0.23 | 0 | 0 | 0.82 | 0.006 | 0.04 | Yes |
| COQ7 | 0.65 | 0 | 0 | 1.18 | 0.005 | 0.037 | No |
| CPA4 | 0.18 | 0.002 | 0.002 | 0.8 | 0.001 | 0.009 | Yes |
| CPM | 3.93 | 0 | 0 | 0.74 | 0 | 0 | No |
| CR2 | 0.71 | 0 | 0 | 0.83 | 0 | 0.003 | Yes |
| CRIP2 | 2.15 | 0 | 0 | 0.78 | 0 | 0.004 | No |
| CRLF1 | 3.12 | 0 | 0 | 0.6 | 0 | 0 | Yes |
| CSF1 | 3.49 | 0 | 0 | 1.29 | 0.004 | 0.032 | No |
| CST6 | -0.61 | 0 | 0 | 0.82 | 0 | 0.002 | Yes |
| CTSB | -1.35 | 0 | 0 | 1.33 | 0 | 0 | No |
| CTSF | -1.22 | 0 | 0 | 1.36 | 0 | 0 | Yes |
| CTSL | 1.54 | 0 | 0 | 1.37 | 0.002 | 0.017 | Yes |
| CTSZ | 2.35 | 0 | 0 | 1.26 | 0.003 | 0.023 | Yes |
| CX3CL1 | -0.52 | 0 | 0 | 1.43 | 0 | 0 | No |
| CXADR | 0.4 | 0 | 0 | 1.22 | 0 | 0 | No |
| CXCL10 | 0.99 | 0 | 0 | 1.13 | 0 | 0.004 | Yes |
| CXCL11 | 0.43 | 0 | 0 | 1.13 | 0 | 0.001 | No |
| CXCL12 | -1.07 | 0 | 0 | 1.42 | 0 | 0 | Yes |
| CXCL8 | 0.12 | 0 | 0 | 1.1 | 0.005 | 0.039 | No |
| CXCL9 | 0.12 | 0 | 0 | 1.26 | 0 | 0 | Yes |
| DCBLD2 | -1.03 | 0 | 0 | 1.35 | 0 | 0.001 | Yes |
| DDC | -1.25 | 0 | 0 | 1.23 | 0 | 0.001 | No |
| DLK1 | 0.99 | 0 | 0 | 0.83 | 0 | 0.001 | No |
| DMP1 | -1.17 | 0 | 0 | 1.57 | 0 | 0 | Yes |
| DPP10 | -1.16 | 0 | 0 | 1.34 | 0 | 0 | No |
| DPP7 | 0.31 | 0 | 0 | 1.16 | 0 | 0.005 | Yes |
| DPT | 4.45 | 0 | 0 | 0.7 | 0 | 0.001 | No |
| DSC2 | 1.08 | 0 | 0 | 1.26 | 0.001 | 0.012 | No |
| DSG2 | -2.44 | 0 | 0 | 1.52 | 0 | 0 | No |
| DSG3 | 0.68 | 0 | 0 | 0.77 | 0 | 0.004 | Yes |
| DUSP13 | 0.42 | 0 | 0 | 0.78 | 0.001 | 0.007 | Yes |
| EBI3_IL27 | -1.05 | 0 | 0 | 1.52 | 0 | 0 | No |
| EDA2R | 0.99 | 0 | 0 | 1.39 | 0 | 0 | Yes |
| EDDM3B | -0.4 | 0 | 0 | 0.83 | 0.004 | 0.029 | Yes |
| ELN | 0.59 | 0 | 0 | 1.29 | 0.001 | 0.013 | Yes |
| ENO3 | 1.49 | 0 | 0 | 0.72 | 0 | 0 | Yes |
| ENTPD5 | -0.4 | 0 | 0 | 1.48 | 0.001 | 0.011 | Yes |
| EPHA2 | 0.9 | 0 | 0 | 1.49 | 0 | 0 | No |
| ESM1 | -2.06 | 0 | 0 | 1.58 | 0 | 0 | No |
| EZR | 3.12 | 0 | 0 | 0.69 | 0 | 0.004 | Yes |
| F10 | 2.24 | 0 | 0 | 0.7 | 0.001 | 0.01 | No |
| F11 | 1.56 | 0 | 0 | 0.75 | 0.005 | 0.034 | No |
| F3 | -1.25 | 0 | 0 | 1.3 | 0.003 | 0.026 | No |
| F7 | 2.73 | 0 | 0 | 0.75 | 0 | 0.003 | No |
| F9 | 6.73 | 0 | 0 | 0.57 | 0 | 0 | Yes |
| FABP4 | 3.88 | 0 | 0 | 0.83 | 0 | 0 | No |
| FAP | 1.39 | 0 | 0 | 0.67 | 0 | 0 | No |
| FCAMR | 1.85 | 0 | 0 | 0.89 | 0.003 | 0.023 | Yes |
| FCGR2B | 0.52 | 0 | 0 | 0.87 | 0.003 | 0.022 | Yes |
| FCRL1 | 1.42 | 0 | 0 | 0.76 | 0 | 0 | Yes |
| FETUB | 1.86 | 0 | 0 | 0.83 | 0.007 | 0.049 | No |
| FGF5 | -1.95 | 0 | 0 | 1.36 | 0 | 0.001 | Yes |
| FGFBP2 | 0.81 | 0 | 0 | 0.76 | 0 | 0 | Yes |
| FGL1 | -0.69 | 0 | 0 | 1.32 | 0 | 0 | Yes |
| FOLR1 | -2.43 | 0 | 0 | 1.34 | 0.001 | 0.006 | No |
| FSHB | -0.5 | 0 | 0 | 1.28 | 0 | 0 | Yes |
| FSTL1 | 1.89 | 0 | 0 | 1.43 | 0.005 | 0.039 | No |
| FURIN | 4.35 | 0 | 0 | 0.76 | 0 | 0.001 | No |
| FUT3_FUT5 | 0.56 | 0 | 0 | 1.32 | 0 | 0 | Yes |
| GALNT10 | 1.41 | 0 | 0 | 1.29 | 0.004 | 0.029 | No |
| GALNT2 | 0.86 | 0 | 0 | 0.76 | 0.005 | 0.035 | Yes |
| GASK1A | 1.98 | 0 | 0 | 0.77 | 0 | 0.001 | No |
| GAST | 0.17 | 0 | 0 | 1.13 | 0 | 0 | Yes |
| GDF15 | 1.13 | 0 | 0 | 1.37 | 0 | 0 | Yes |
| GDF2 | -1.72 | 0 | 0 | 1.31 | 0 | 0.001 | Yes |
| GFAP | -0.86 | 0 | 0 | 1.16 | 0.004 | 0.033 | No |
| GH1 | -0.41 | 0 | 0 | 1.06 | 0 | 0 | No |
| GHR | 5.53 | 0 | 0 | 0.42 | 0 | 0 | No |
| GRN | 2.03 | 0 | 0 | 1.35 | 0.003 | 0.024 | Yes |
| GRPEL1 | 0.86 | 0 | 0 | 1.17 | 0 | 0.001 | No |
| GSTA1 | 1.54 | 0 | 0 | 0.88 | 0 | 0 | No |
| GSTA3 | 1.55 | 0 | 0 | 0.89 | 0 | 0.004 | No |
| GUCA2A | -1.7 | 0 | 0 | 1.31 | 0 | 0.003 | No |
| GZMA | 2.06 | 0 | 0 | 0.81 | 0.003 | 0.026 | No |
| GZMB | 0.13 | 0 | 0 | 0.86 | 0 | 0.002 | Yes |
| HAVCR1 | 0.93 | 0 | 0 | 1.16 | 0 | 0.002 | Yes |
| HJV | 2.27 | 0 | 0 | 0.79 | 0.001 | 0.008 | Yes |
| HLA_E | 1.26 | 0 | 0 | 1.26 | 0.006 | 0.041 | No |
| HMOX1 | 0.25 | 0 | 0 | 1.21 | 0 | 0.003 | Yes |
| HPGDS | 0.42 | 0 | 0 | 0.71 | 0 | 0 | No |
| HSD11B1 | -2.62 | 0 | 0 | 1.49 | 0 | 0 | Yes |
| HSDL2 | 1.14 | 0 | 0 | 0.8 | 0 | 0.005 | Yes |
| ICAM5 | -0.97 | 0 | 0 | 1.19 | 0.002 | 0.014 | No |
| IDO1 | 0.75 | 0 | 0 | 0.87 | 0.003 | 0.023 | Yes |
| IFI30 | 2.2 | 0 | 0 | 1.26 | 0.001 | 0.012 | No |
| IGDCC4 | -1.77 | 0 | 0 | 0.59 | 0 | 0 | Yes |
| IGFBP1 | -1.11 | 0 | 0 | 1.17 | 0 | 0 | No |
| IGFBP2 | -2.56 | 0 | 0 | 1.55 | 0 | 0 | No |
| IGFBP3 | 0.18 | 0.012 | 0.014 | 0.66 | 0 | 0 | Yes |
| IGFBP6 | 2.47 | 0 | 0 | 0.68 | 0 | 0 | No |
| IGFBP7 | 1.27 | 0 | 0 | 1.25 | 0.003 | 0.023 | No |
| IGSF9 | 2.13 | 0 | 0 | 0.83 | 0 | 0 | No |
| IL10RA | 0.21 | 0 | 0 | 1.11 | 0.006 | 0.041 | Yes |
| IL13RA1 | 3.93 | 0 | 0 | 0.72 | 0.003 | 0.024 | No |
| IL15 | 0.2 | 0.002 | 0.003 | 1.51 | 0 | 0 | No |
| IL17RB | -1.59 | 0 | 0 | 1.22 | 0 | 0 | No |
| IL18BP | 1.09 | 0 | 0 | 1.26 | 0.003 | 0.024 | Yes |
| IL1R1 | -1.73 | 0 | 0 | 1.64 | 0 | 0 | No |
| IL1RL1 | 0.18 | 0 | 0 | 1.22 | 0 | 0.003 | Yes |
| IL1RN | 2.97 | 0 | 0 | 0.89 | 0.005 | 0.038 | No |
| IL2RA | 1.11 | 0 | 0 | 1.34 | 0 | 0 | No |
| IL32 | -0.82 | 0 | 0 | 1.45 | 0 | 0 | Yes |
| IL4R | 0.31 | 0 | 0 | 1.35 | 0 | 0 | No |
| INHBC | 3.28 | 0 | 0 | 0.82 | 0 | 0.002 | Yes |
| ITIH3 | -1.03 | 0 | 0 | 1.37 | 0 | 0 | No |
| KDR | 1.99 | 0 | 0 | 0.73 | 0.001 | 0.011 | No |
| KIAA0319 | -2.66 | 0 | 0 | 1.32 | 0.001 | 0.01 | No |
| KIRREL2 | -1.23 | 0 | 0 | 1.28 | 0 | 0.001 | No |
| KLB | -0.62 | 0 | 0 | 1.14 | 0 | 0.001 | Yes |
| KLK4 | -0.35 | 0 | 0 | 1.14 | 0.001 | 0.006 | Yes |
| KLK8 | 1.2 | 0 | 0 | 0.76 | 0 | 0.002 | Yes |
| KLKB1 | 2.37 | 0 | 0 | 0.63 | 0 | 0.001 | Yes |
| KRT19 | -0.17 | 0 | 0 | 1.2 | 0 | 0 | No |
| LAMP3 | 0.29 | 0 | 0 | 1.21 | 0 | 0 | No |
| LAYN | -0.39 | 0 | 0 | 1.39 | 0 | 0 | No |
| LCAT | 5.52 | 0 | 0 | 0.53 | 0 | 0 | No |
| LDLR | 2.28 | 0 | 0 | 0.73 | 0 | 0 | No |
| LEP | 3.02 | 0 | 0 | 0.82 | 0 | 0 | Yes |
| LEPR | -3.28 | 0 | 0 | 1.5 | 0 | 0 | No |
| LGALS1 | 3.23 | 0 | 0 | 0.8 | 0.001 | 0.012 | No |
| LGALS4 | 0.78 | 0 | 0 | 1.16 | 0.001 | 0.009 | No |
| LGMN | 1.08 | 0 | 0 | 1.23 | 0.002 | 0.018 | No |
| LPL | -1.13 | 0 | 0 | 1.2 | 0.001 | 0.009 | Yes |
| LRCH4 | 0.82 | 0 | 0 | 0.88 | 0.005 | 0.035 | No |
| LRP1 | -1.69 | 0 | 0 | 1.63 | 0 | 0 | Yes |
| LRRN1 | -0.78 | 0 | 0 | 0.73 | 0 | 0 | Yes |
| LTB | 0.72 | 0 | 0 | 0.72 | 0 | 0.005 | No |
| LTBP2 | -1.97 | 0 | 0 | 1.52 | 0 | 0 | No |
| LTBR | 1.65 | 0 | 0 | 1.43 | 0 | 0.001 | No |
| LUZP2 | -0.81 | 0 | 0 | 1.19 | 0.002 | 0.019 | Yes |
| MANSC1 | -0.68 | 0 | 0 | 1.48 | 0 | 0 | No |
| MASP1 | 2.28 | 0 | 0 | 0.71 | 0.001 | 0.013 | Yes |
| MATN3 | -1.67 | 0 | 0 | 1.3 | 0 | 0.006 | No |
| MBL2 | -0.26 | 0 | 0 | 1.1 | 0.001 | 0.009 | No |
| MDK | 0.22 | 0 | 0 | 1.13 | 0.006 | 0.04 | Yes |
| MEGF9 | 3.87 | 0 | 0 | 0.62 | 0 | 0.001 | No |
| MENT | -3.09 | 0 | 0 | 1.67 | 0 | 0.002 | Yes |
| MEP1B | 0.52 | 0 | 0 | 0.92 | 0 | 0.001 | No |
| MEPE | 0.45 | 0 | 0 | 0.75 | 0.002 | 0.017 | Yes |
| MFAP5 | 2.86 | 0 | 0 | 0.71 | 0 | 0 | No |
| MMP13 | -1.03 | 0 | 0 | 1.38 | 0 | 0 | No |
| MMP3 | -0.59 | 0 | 0 | 1.22 | 0 | 0.001 | No |
| MOG | -2.47 | 0 | 0 | 1.26 | 0.002 | 0.016 | No |
| MSLN | -0.43 | 0 | 0 | 1.17 | 0 | 0.002 | No |
| MSTN | 0.45 | 0 | 0 | 0.77 | 0 | 0 | No |
| MTHFD2 | -0.61 | 0 | 0 | 1.41 | 0 | 0.002 | Yes |
| MXRA8 | -3.65 | 0 | 0 | 1.45 | 0 | 0.001 | No |
| NAAA | -0.61 | 0 | 0 | 1.15 | 0.001 | 0.007 | No |
| NCAM2 | -3.63 | 0 | 0 | 1.52 | 0 | 0 | Yes |
| NCR3LG1 | -0.57 | 0 | 0 | 1.39 | 0 | 0.002 | No |
| NECTIN2 | 1.51 | 0 | 0 | 1.42 | 0 | 0 | Yes |
| NEDD4L | 0.31 | 0 | 0 | 0.83 | 0.002 | 0.014 | Yes |
| NEFL | -1.41 | 0 | 0 | 1.37 | 0 | 0 | Yes |
| NFX1 | 0.41 | 0 | 0 | 0.89 | 0.006 | 0.044 | No |
| NID1 | 0.81 | 0 | 0 | 1.18 | 0.007 | 0.046 | No |
| NINJ1 | 0.12 | 0.001 | 0.002 | 1.12 | 0.004 | 0.03 | No |
| NPY | 0.68 | 0 | 0 | 0.88 | 0 | 0.005 | No |
| NRP1 | -0.29 | 0 | 0 | 1.43 | 0 | 0.001 | Yes |
| NTPROBNP | -0.24 | 0 | 0 | 1.07 | 0.002 | 0.017 | Yes |
| NTRK2 | 2.33 | 0 | 0 | 0.63 | 0 | 0 | No |
| NXPH1 | -1.06 | 0 | 0 | 1.22 | 0.001 | 0.013 | Yes |
| OPTC | -1.93 | 0 | 0 | 1.24 | 0 | 0 | Yes |
| OSCAR | 1 | 0 | 0 | 1.32 | 0 | 0.003 | No |
| OSMR | 1 | 0 | 0 | 1.54 | 0 | 0.003 | Yes |
| OXT | 0.93 | 0 | 0 | 0.89 | 0 | 0 | Yes |
| PAEP | -0.07 | 0 | 0 | 0.93 | 0.002 | 0.014 | Yes |
| PAG1 | 1.21 | 0 | 0 | 0.87 | 0.004 | 0.028 | No |
| PALM | 4.53 | 0 | 0 | 0.68 | 0 | 0 | Yes |
| PAMR1 | 4.39 | 0 | 0 | 0.62 | 0 | 0 | No |
| PAPPA | -0.25 | 0 | 0 | 1.21 | 0 | 0.005 | No |
| PDCD1 | 0.47 | 0 | 0 | 1.22 | 0 | 0.003 | No |
| PDCD1LG2 | -0.28 | 0 | 0 | 1.28 | 0.002 | 0.014 | No |
| PGA4 | -0.47 | 0 | 0 | 1.2 | 0 | 0 | Yes |
| PGF | 3.09 | 0 | 0 | 1.32 | 0.003 | 0.027 | No |
| PIGR | 0.64 | 0 | 0 | 1.42 | 0 | 0 | No |
| PILRA | 1.4 | 0 | 0 | 1.19 | 0.003 | 0.025 | No |
| PLA2G10 | -1.29 | 0 | 0 | 1.18 | 0 | 0.001 | Yes |
| PLA2G15 | 2.56 | 0 | 0 | 1.6 | 0 | 0 | Yes |
| PLAT | 2.57 | 0 | 0 | 0.87 | 0.003 | 0.023 | No |
| PLAUR | 1.87 | 0 | 0 | 1.64 | 0 | 0 | Yes |
| PLTP | -2.38 | 0 | 0 | 1.42 | 0 | 0 | No |
| PM20D1 | 0.02 | 0.034 | 0.041 | 0.95 | 0 | 0.001 | Yes |
| PODXL2 | -2.44 | 0 | 0 | 1.31 | 0.002 | 0.017 | Yes |
| POMC | 0.62 | 0 | 0 | 0.86 | 0 | 0.003 | No |
| PON3 | -4.79 | 0 | 0 | 1.6 | 0 | 0 | No |
| PRAP1 | 3.92 | 0 | 0 | 0.75 | 0 | 0 | Yes |
| PRELP | 0.79 | 0 | 0 | 0.69 | 0 | 0.002 | No |
| PRG2 | 0.36 | 0 | 0 | 1.21 | 0 | 0.004 | Yes |
| PROK1 | 0.31 | 0 | 0 | 0.84 | 0 | 0.001 | Yes |
| PROS1 | 3.17 | 0 | 0 | 0.68 | 0.001 | 0.006 | No |
| PRRT3 | -2.61 | 0 | 0 | 1.37 | 0 | 0 | No |
| PSG1 | -0.24 | 0 | 0 | 1.13 | 0 | 0 | Yes |
| PSRC1 | 0.58 | 0 | 0 | 0.87 | 0.002 | 0.014 | Yes |
| PTPRN2 | -2.03 | 0 | 0 | 1.34 | 0 | 0.001 | Yes |
| PTPRR | -1.93 | 0 | 0 | 1.25 | 0.004 | 0.033 | No |
| PTPRZ1 | -2.81 | 0 | 0 | 1.54 | 0 | 0 | No |
| PTX3 | -0.58 | 0 | 0 | 1.2 | 0 | 0.006 | Yes |
| PVR | 0.51 | 0 | 0 | 1.21 | 0.003 | 0.025 | No |
| RBP2 | 0.17 | 0 | 0 | 1.13 | 0 | 0.002 | No |
| REG1A | -0.75 | 0 | 0 | 1.21 | 0 | 0.001 | No |
| REG1B | -0.39 | 0 | 0 | 1.12 | 0.004 | 0.028 | Yes |
| REG3A | -0.55 | 0 | 0 | 1.14 | 0.001 | 0.006 | Yes |
| REN | 0.35 | 0 | 0 | 1.13 | 0 | 0.003 | Yes |
| RET | 2.27 | 0 | 0 | 0.63 | 0 | 0 | Yes |
| RNASET2 | 2.04 | 0 | 0 | 1.32 | 0.002 | 0.02 | No |
| RSPO1 | -0.5 | 0 | 0 | 1.36 | 0 | 0 | Yes |
| RTN4R | 3.82 | 0 | 0 | 0.61 | 0 | 0 | Yes |
| S100A13 | 1.18 | 0 | 0 | 0.78 | 0.004 | 0.03 | No |
| SCARF2 | -1.56 | 0 | 0 | 1.46 | 0 | 0 | Yes |
| SCG3 | -2.65 | 0 | 0 | 1.28 | 0.001 | 0.007 | Yes |
| SCGB3A1 | -3.05 | 0 | 0 | 1.63 | 0 | 0 | No |
| SCGB3A2 | -1.17 | 0 | 0 | 1.12 | 0 | 0 | No |
| SCGN | -0.91 | 0 | 0 | 1.2 | 0.001 | 0.01 | No |
| SELENOP | -0.4 | 0 | 0 | 0.63 | 0 | 0.004 | Yes |
| SELPLG | 1.62 | 0 | 0 | 0.73 | 0 | 0.002 | No |
| SERPINA1 | -0.79 | 0.01 | 0.013 | 3.92 | 0 | 0 | Yes |
| SERPINA11 | -0.92 | 0 | 0 | 1.53 | 0 | 0 | Yes |
| SERPINA3 | 1.84 | 0 | 0 | 1.88 | 0.001 | 0.006 | No |
| SERPINA4 | 1.05 | 0 | 0 | 0.64 | 0 | 0.003 | Yes |
| SERPINA5 | 2.45 | 0 | 0 | 0.69 | 0 | 0.001 | Yes |
| SERPINF1 | 6.04 | 0 | 0 | 0.61 | 0 | 0 | No |
| SERPINF2 | 3.91 | 0 | 0 | 0.52 | 0.002 | 0.015 | No |
| SEZ6L | -3.58 | 0 | 0 | 1.3 | 0.003 | 0.024 | No |
| SFRP4 | 2.26 | 0 | 0 | 0.56 | 0 | 0 | Yes |
| SHBG | -2.67 | 0 | 0 | 1.55 | 0 | 0 | No |
| SIT1 | 1.32 | 0 | 0 | 0.83 | 0 | 0.001 | Yes |
| SKAP1 | 0.92 | 0 | 0 | 0.87 | 0 | 0.001 | No |
| SLAMF8 | 0.6 | 0 | 0 | 1.24 | 0 | 0 | No |
| SLC39A14 | -1.72 | 0 | 0 | 1.35 | 0 | 0 | No |
| SLITRK1 | -3.87 | 0 | 0 | 1.33 | 0.001 | 0.011 | Yes |
| SLITRK2 | 1.89 | 0 | 0 | 0.75 | 0 | 0 | Yes |
| SLITRK6 | -1.15 | 0 | 0 | 1.22 | 0.007 | 0.045 | No |
| SLURP1 | 1.04 | 0 | 0 | 0.8 | 0.007 | 0.046 | Yes |
| SMAD5 | 3.18 | 0 | 0 | 0.64 | 0 | 0.002 | No |
| SMOC2 | 1.73 | 0 | 0 | 0.75 | 0 | 0 | Yes |
| SMPDL3A | -0.46 | 0 | 0 | 1.19 | 0 | 0 | No |
| SORD | 1.25 | 0 | 0 | 0.9 | 0.005 | 0.035 | No |
| SOST | 1.49 | 0 | 0 | 0.45 | 0 | 0 | Yes |
| SPINK1 | -0.35 | 0 | 0 | 1.36 | 0 | 0 | Yes |
| SPINK5 | -0.65 | 0 | 0 | 0.79 | 0.002 | 0.018 | Yes |
| SPINK6 | 1.09 | 0 | 0 | 0.87 | 0.003 | 0.023 | No |
| SPON1 | 0.25 | 0 | 0 | 1.38 | 0 | 0 | Yes |
| SPON2 | 1.99 | 0 | 0 | 1.26 | 0.001 | 0.012 | No |
| SPP1 | -0.57 | 0 | 0 | 1.5 | 0 | 0 | No |
| SRPX | -1.02 | 0 | 0 | 1.45 | 0 | 0 | No |
| SSC4D | 1.28 | 0 | 0 | 0.85 | 0 | 0 | No |
| SSC5D | 2.49 | 0 | 0 | 0.83 | 0.001 | 0.009 | No |
| STC2 | 2.67 | 0 | 0 | 0.7 | 0 | 0.002 | No |
| STX7 | 0.77 | 0 | 0 | 0.79 | 0 | 0.001 | Yes |
| SULT2A1 | 1.29 | 0 | 0 | 1.13 | 0.004 | 0.029 | Yes |
| SUSD2 | -2.23 | 0 | 0 | 1.38 | 0 | 0.001 | No |
| SUSD5 | -1.45 | 0 | 0 | 1.32 | 0.001 | 0.013 | No |
| TCL1A | 0.51 | 0 | 0 | 0.9 | 0 | 0 | No |
| TFF2 | -0.28 | 0 | 0 | 1.26 | 0 | 0 | No |
| TGFBR3 | -0.46 | 0 | 0 | 1.26 | 0.003 | 0.024 | No |
| THBS4 | 2.34 | 0 | 0 | 0.82 | 0 | 0.002 | Yes |
| TIGIT | 0.58 | 0 | 0 | 1.24 | 0.003 | 0.023 | Yes |
| TIMP4 | 0.92 | 0 | 0 | 1.35 | 0 | 0 | Yes |
| TNC | -0.43 | 0 | 0 | 1.29 | 0 | 0 | No |
| TNF | 1.31 | 0 | 0 | 1.31 | 0 | 0 | Yes |
| TNFRSF10A | 2.44 | 0 | 0 | 1.27 | 0.001 | 0.01 | No |
| TNFRSF10B | 1.52 | 0 | 0 | 1.17 | 0.001 | 0.014 | No |
| TNFRSF11B | 0.52 | 0 | 0 | 1.32 | 0.001 | 0.009 | No |
| TNFRSF1B | 2.07 | 0 | 0 | 1.28 | 0 | 0 | No |
| TNFRSF4 | 1.34 | 0 | 0 | 1.39 | 0 | 0 | Yes |
| TNFRSF6B | 1.28 | 0 | 0 | 1.16 | 0 | 0.004 | Yes |
| TNFRSF9 | 1.08 | 0 | 0 | 1.28 | 0 | 0 | No |
| TNFSF11 | 0.88 | 0 | 0 | 0.8 | 0 | 0 | Yes |
| TPP1 | 1.3 | 0 | 0 | 1.17 | 0.002 | 0.016 | No |
| TREH | 0.96 | 0 | 0 | 0.89 | 0.001 | 0.014 | No |
| TRIM21 | 0.3 | 0 | 0 | 0.91 | 0.001 | 0.013 | No |
| TSHB | 0.25 | 0 | 0 | 0.89 | 0 | 0.002 | Yes |
| TTR | 0.24 | 0.014 | 0.017 | 0.59 | 0 | 0 | Yes |
| UNC5D | -2.13 | 0 | 0 | 1.45 | 0.001 | 0.006 | Yes |
| UPB1 | 1.37 | 0 | 0 | 0.9 | 0.001 | 0.01 | No |
| VAMP5 | 0.37 | 0 | 0 | 1.2 | 0 | 0 | Yes |
| VCAM1 | 0.28 | 0 | 0.001 | 1.45 | 0 | 0 | No |
| VCAN | -1.55 | 0 | 0 | 1.72 | 0 | 0 | Yes |
| VEGFA | 1.01 | 0 | 0 | 1.12 | 0.005 | 0.037 | Yes |
| VGF | -2.48 | 0 | 0 | 1.34 | 0.001 | 0.013 | No |
| VNN1 | 1.41 | 0 | 0 | 0.86 | 0 | 0.001 | No |
| WDR46 | 1.4 | 0 | 0 | 0.78 | 0.004 | 0.033 | No |
| WFDC2 | -0.62 | 0 | 0 | 1.46 | 0 | 0 | No |
| WFIKKN1 | 1.45 | 0 | 0 | 0.71 | 0 | 0 | Yes |
| WNT9A | -1.42 | 0 | 0 | 1.34 | 0 | 0.005 | Yes |
| XCL1 | 0.83 | 0 | 0 | 1.12 | 0.006 | 0.044 | Yes |
| XG | 2.62 | 0 | 0 | 0.72 | 0 | 0 | Yes |
| ZBTB17 | 0.92 | 0 | 0 | 1.17 | 0.005 | 0.034 | Yes |

**Table S7 Mediation analysis of blood proteins on adult BMI-OP**

| Exposures | Mediators | Outcome | PM (95% CI) | P | FDR |
| --- | --- | --- | --- | --- | --- |
| adult BMI | ADIPOQ | OP | 25.6 (17.9, 33.8) | 0 | 0 |
| adult BMI | SOST | OP | 16.7 (12.2, 21) | 0 | 0 |
| adult BMI | SFRP4 | OP | 15.8 (10.2, 21.8) | 0 | 0 |
| adult BMI | CDHR5 | OP | 14.6 (10, 21) | 0 | 0 |
| adult BMI | RTN4R | OP | 13.9 (7.9, 24.7) | 0 | 0 |
| adult BMI | DMP1 | OP | 13.8 (9.5, 19.5) | 0 | 0 |
| adult BMI | CCN5 | OP | 13.7 (8.5, 20.6) | 0 | 0 |
| adult BMI | OXT | OP | 12.4 (5.4, 21.3) | 0 | 0 |
| adult BMI | RET | OP | 11.9 (7.4, 16.8) | 0 | 0 |
| adult BMI | ENO3 | OP | 10.9 (6.3, 16.7) | 0 | 0 |
| adult BMI | HSD11B1 | OP | 10.4 (5, 16) | 0 | 0 |
| adult BMI | CRLF1 | OP | 7.2 (3.1, 12.1) | 0 | 0 |
| adult BMI | NEFL | OP | 7.1 (4.1, 10) | 0 | 0 |
| adult BMI | VCAN | OP | 6.6 (4.2, 10.2) | 0 | 0 |
| adult BMI | NCAM2 | OP | 6.6 (1.7, 12.3) | 0 | 0 |
| adult BMI | WFIKKN1 | OP | 6.5 (3.4, 9.6) | 0 | 0 |
| adult BMI | FGL1 | OP | 5.9 (3.9, 9.5) | 0 | 0 |
| adult BMI | TNFSF11 | OP | 5.6 (2.9, 8.2) | 0 | 0 |
| adult BMI | OPTC | OP | 5.5 (1, 9) | 0.04 | 0.04712329 |
| adult BMI | SERPINA11 | OP | 5.4 (3.7, 8) | 0 | 0 |
| adult BMI | CDA | OP | 5.4 (1.3, 9.4) | 0.02 | 0.02548148 |
| adult BMI | SLITRK2 | OP | 5.4 (1.2, 9.1) | 0.04 | 0.04712329 |
| adult BMI | CNDP1 | OP | 5.4 (2.5, 7.7) | 0 | 0 |
| adult BMI | FCRL1 | OP | 5.2 (2.1, 9.3) | 0 | 0 |
| adult BMI | IL32 | OP | 5 (2.6, 7.4) | 0 | 0 |
| adult BMI | CXCL12 | OP | 4.9 (2.8, 7) | 0 | 0 |
| adult BMI | MENT | OP | 4.9 (1.4, 8.4) | 0 | 0 |
| adult BMI | LRP1 | OP | 4.8 (2.1, 7.2) | 0 | 0 |
| adult BMI | SMOC2 | OP | 4.7 (2.1, 9.4) | 0.02 | 0.02548148 |
| adult BMI | SERPINA5 | OP | 4.6 (0.8, 8.3) | 0.04 | 0.04712329 |
| adult BMI | PLA2G10 | OP | 4.5 (0.1, 9.4) | 0.04 | 0.04712329 |
| adult BMI | CTSF | OP | 4.5 (2.4, 6.8) | 0 | 0 |
| adult BMI | CALB1 | OP | 4.3 (1.3, 7.2) | 0 | 0 |
| adult BMI | XG | OP | 4.3 (1, 9) | 0.02 | 0.02548148 |
| adult BMI | CBLN4 | OP | 4.2 (2.9, 5.8) | 0 | 0 |
| adult BMI | SIT1 | OP | 4.2 (0.8, 8.6) | 0.04 | 0.04712329 |
| adult BMI | FGFBP2 | OP | 4 (1.9, 7.3) | 0 | 0 |
| adult BMI | KLB | OP | 3.9 (1.7, 6.5) | 0 | 0 |
| adult BMI | KLKB1 | OP | 3.9 (1.6, 6.9) | 0 | 0 |
| adult BMI | CHRDL2 | OP | 3.8 (2, 5.6) | 0 | 0 |
| adult BMI | CA9 | OP | 3.8 (0.8, 6.8) | 0 | 0 |
| adult BMI | FGF5 | OP | 3.7 (1.2, 7) | 0.02 | 0.02548148 |
| adult BMI | SCARF2 | OP | 3.6 (1.4, 6.4) | 0 | 0 |
| adult BMI | CHL1 | OP | 3.5 (1, 5.8) | 0.02 | 0.02548148 |
| adult BMI | GDF2 | OP | 3.4 (0.4, 6.4) | 0.04 | 0.04712329 |
| adult BMI | FSHB | OP | 3.3 (1.7, 5.2) | 0 | 0 |
| adult BMI | PSG1 | OP | 3.2 (1.8, 4.6) | 0 | 0 |
| adult BMI | UNC5D | OP | 3.2 (0.8, 5.9) | 0 | 0 |
| adult BMI | PGA4 | OP | 3.1 (1.5, 4.7) | 0 | 0 |
| adult BMI | HSDL2 | OP | 3.1 (0.5, 5.4) | 0 | 0 |
| adult BMI | AHSG | OP | 3.1 (1.4, 6.5) | 0 | 0 |
| adult BMI | MASP1 | OP | 3 (1.1, 5.6) | 0 | 0 |
| adult BMI | DCBLD2 | OP | 3 (1.2, 4.5) | 0 | 0 |
| adult BMI | STX7 | OP | 3 (1.1, 4.7) | 0.02 | 0.02548148 |
| adult BMI | LPL | OP | 3 (0.4, 6.2) | 0.02 | 0.02548148 |
| adult BMI | CEBPB | OP | 2.7 (0.5, 4.9) | 0.02 | 0.02548148 |
| adult BMI | AGR2 | OP | 2.6 (1.5, 4.2) | 0 | 0 |
| adult BMI | CLEC5A | OP | 2.5 (1.5, 4) | 0 | 0 |
| adult BMI | KLK8 | OP | 2.4 (0.5, 5.4) | 0.02 | 0.02548148 |
| adult BMI | WNT9A | OP | 2.4 (0.9, 4.6) | 0 | 0 |
| adult BMI | CR2 | OP | 2.3 (0.5, 4) | 0.02 | 0.02548148 |
| adult BMI | LUZP2 | OP | 2 (0.4, 4) | 0.02 | 0.02548148 |
| adult BMI | SERPINA4 | OP | 2 (0.7, 3.7) | 0 | 0 |
| adult BMI | REG3A | OP | 2 (0.4, 4) | 0 | 0 |
| adult BMI | PTX3 | OP | 1.9 (0.5, 3.5) | 0.02 | 0.02548148 |
| adult BMI | CGA | OP | 1.9 (1, 3.1) | 0 | 0 |
| adult BMI | SPINK1 | OP | 1.9 (1.1, 2.7) | 0 | 0 |
| adult BMI | FCGR2B | OP | 1.8 (0.4, 3.1) | 0.04 | 0.04712329 |
| adult BMI | KLK4 | OP | 1.7 (0.6, 2.9) | 0 | 0 |
| adult BMI | RSPO1 | OP | 1.7 (0.8, 2.8) | 0 | 0 |
| adult BMI | PROK1 | OP | 1.6 (0.7, 2.7) | 0 | 0 |
| adult BMI | DUSP13 | OP | 1.6 (0.5, 2.6) | 0 | 0 |
| adult BMI | DSG3 | OP | 1.6 (0.5, 2.7) | 0.02 | 0.02548148 |
| adult BMI | TSHB | OP | 1.4 (0.6, 2.3) | 0 | 0 |
| adult BMI | NTPROBNP | OP | 1.3 (0.1, 2.6) | 0.04 | 0.04712329 |
| adult BMI | GALNT2 | OP | 1.3 (0.3, 2.7) | 0 | 0 |
| adult BMI | REG1B | OP | 1.3 (0.2, 2.5) | 0.04 | 0.04712329 |
| adult BMI | COL1A1 | OP | 1.2 (0.1, 2.3) | 0.04 | 0.04712329 |
| adult BMI | MTHFD2 | OP | 1.1 (0.4, 2) | 0 | 0 |
| adult BMI | NEDD4L | OP | 0.9 (0.2, 1.8) | 0.02 | 0.02548148 |
| adult BMI | CKMT1A_CKMT1B | OP | 0.9 (0.1, 1.6) | 0.02 | 0.02548148 |
| adult BMI | C1QA | OP | 0.9 (0.3, 1.4) | 0 | 0 |
| adult BMI | GZMB | OP | 0.8 (0.3, 1.4) | 0 | 0 |
| adult BMI | NRP1 | OP | 0.7 (0.3, 1.4) | 0 | 0 |
| adult BMI | CEP170 | OP | 0.7 (0.2, 1.6) | 0.02 | 0.02548148 |
| adult BMI | TTR | OP | 0.7 (0.1, 1.5) | 0 | 0 |
| adult BMI | COPB2 | OP | 0.7 (0.1, 1.3) | 0 | 0 |
| adult BMI | SERPINA1 | OP | 0.6 (0.2, 1.3) | 0 | 0 |
| adult BMI | ENTPD5 | OP | 0.6 (0.1, 1.3) | 0 | 0 |
| adult BMI | MEPE | OP | 0.6 (0.1, 1.2) | 0.02 | 0.02548148 |
| adult BMI | IGFBP3 | OP | 0.6 (0.2, 1.4) | 0 | 0 |
| adult BMI | CPA4 | OP | 0.5 (0.1, 1.1) | 0 | 0 |
| adult BMI | CLSTN2 | OP | 0.4 (0.1, 1) | 0.02 | 0.02548148 |
| adult BMI | PM20D1 | OP | 0.4 (0, 0.9) | 0.04 | 0.04712329 |
| adult BMI | ARNT | OP | 0.3 (0.1, 0.6) | 0 | 0 |

**Figure S1: Associations between life course adiposity and incident OP adjusted for model 1 (A) and model 2 (B).**

Non-linear relationships were estimated using restricted cubic splines and linear relationships were estimated using Cox-proportional hazards regression analysis. The p values for non-linear and linear associations are listed in the figures for all outcomes. Dashed vertical lines in the left and right-hand panel represent WHO birth weight category thresholds of 2.5 kg (low to normal birth weight), 4 kg (normal to high birth weight), and BMI category thresholds of 18.5 kg/m² (underweight to normal weight), 25 kg/m² (normal weight to overweight), 30 kg/m² (overweight to obesity).
